# *Moringa oleifera* leaf polysaccharide alleviates experimental colitis by inhibiting inflammation and maintaining intestinal barrier

**DOI:** 10.3389/fnut.2022.1055791

**Published:** 2022-11-10

**Authors:** Hosameldeen Mohamed Husien, WeiLong Peng, Hongrui Su, RuiGang Zhou, Ya Tao, JunJie Huang, MingJiang Liu, RuoNan Bo, JinGui Li

**Affiliations:** ^1^College of Veterinary Medicine, Yangzhou University, Yangzhou, China; ^2^Jiangsu Co-innovation Center for Prevention and Control of Important Animal Infectious Diseases and Zoonoses, Yangzhou, China; ^3^College of Veterinary Medicine, University of Albutana, Albutana, Sudan

**Keywords:** inflammatory bowel disease, *Moringa oleifera* leaves polysaccharide, intestinal injury, inflammatory signaling pathway, tight junction expression

## Abstract

The characteristic of ulcerative colitis (UC) is extensive colonic mucosal inflammation. *Moringa oleifera* (*M. oleifera*) is a medicine food homology plant, and the polysaccharide from *M. oleifera* leaves (MOLP) exhibits antioxidant and anti-inflammatory activity. The aim of this study to investigate the potential effect of MOLP on UC in a mouse model as well as the underlying mechanism. Dextran sulfate sodium (DSS) 4% in drinking water was given for 7 days to mice with UC, at the same time, MOLP (25, 50, and 100 mg/kg/day) was intragastric administered once daily during the experiment. Structural analysis revealed that MOLP had an average molecular weight (Mw) of 182,989 kDa and consisted of fucose, arabinose, rhamnose, galactose, glucose, xylose, mannose, galactose uronic acid, glucuronic acid, glucose uronic acid and mannose uronic acid, with a percentage ratio of 1.64, 18.81, 12.04, 25.90, 17.57, 12.01, 3.51, 5.28, 0.55, 1.27, and 1.43%, respectively. In addition, the features of MOLP were identified by Fourier-transform infrared (FT-IR) and spectra, X-ray diffraction (XRD). The results showed that MOLP exhibited protective efficacy against UC by alleviating colonic pathological alterations, decreasing goblet cells, crypt destruction, and infiltration of inflammatory cells caused by DSS. Furthermore, MOLP notably repressed the loss of zonula occludens-1 (ZO-1) and occludin proteins in mucosal layer, as well as up-regulating the mRNA expression of interleukin-10 (IL-10) and peroxisome proliferator-activated receptor-γ (PPAR-γ), whereas down-regulating the activation of Toll-like receptor 4 (TLR4), myeloid differentiation primary response 88 (MyD88), nuclear factor-kappa B (NF-κB) signaling pathway and the production of pro-inflammatory cytokines. Therefore, these results will help understand the protective action procedure of MOLP against UC, thereby providing significance for the development of MOLP.

## Introduction

Ulcerative colitis (UC), a common consequence of inflammatory bowel disease (IBD), is a chronic recurring intestinal disease, indicated by abdominal pain, losing weight, and bloody stools (1). Recently, anti-inflammatory drugs (sulfasalazine or mesalazine), common immunosuppressive agents (glucocorticoids, azathioprine, methotrexate, and cyclosporine A), and biological products, such as infliximab and adalimumab, have been shown to help relieve UC symptoms (2, 3). Nevertheless, these treatments are restricted due to their side effects or serious adverse events, such as steroid dependence and secondary infection (4). Consequently, developing effective alternative strategies for preventing and mitigating UC is critical.

Damage to the intestinal mucosal barrier allows external antigens (LPS et al.) and pathogens to infiltrate and activate immune cells in the body’s lamina propria. The activated immune cells then initiate an inflammatory cascade marked by elevated levels of pro-inflammatory cytokines such interleukin-1β (IL-1β) and tumor necrosis factor alpha (TNF-α), as well as a reduction in the anti-inflammatory cytokine interleukin-10 (IL-10) (5). Pro-inflammatory factors motivate macrophage and neutrophil infiltration, stimulate mucosal permeability, and reduce tight junction (TJ) proteins, ultimately leading to tissue injury (6).

*Moringa oleifera* (*M. oleifera*), a perennial plant with considerable nutritional and medicinal benefits, is a component of the Moringa family. It grows widely throughout Southeast Asia, Africa, China’s southern region, and even the rest of the world (7). The World Health Organization (WHO) has recommended *M. oleifera* leaves as a highly nutritious alternative to imported food sources for the treatment of malnutrition (8). The leaves are healthful whether eaten fresh or cooked. Furthermore, *M. oleifera* leaf extracts have a variety of biological characteristics, such as antioxidants activity, hypoglycemic activity, anti-inflammatory activity, and immunomodulatory activity (9, 10).

Recent research indicates that polysaccharides extracted from *M. oleifera* leaves have acquired popularity due to their diverse and excellent biological activity. For example, a novel arabinogalactan (MOP-1) with considerable *in vitro* antioxidant activity was extracted from the leaves of *M. oleifera* (11). Dong et al. obtained another polysaccharide (MOP-2) from the leaves of *M. oleifera* and measured its immunomodulatory activity *in vitro* (12). However, the potential effect of *M. oleifera* leaf polysaccharide (MOLP) against UC and the underlying mechanism unclear.

In this study, we examine the potential protective effect of MOLP on dextran sulfate sodium (DSS)-induced UC in mice by detecting colonic histopathological alterations, Toll-like receptor 4 (TLR4), Myeloid differentiation primary response 88 (MyD88), Nuclear factor-kappa B (NF-κB) signaling pathways and the corresponding inflammatory cytokines, TNF-α, high mobility group box 1 (HMGB1), peroxisome proliferator-activated receptor-γ (PPAR-γ) and so on. In addition, mucosal permeation related TJ protein levels were also analyzed. These findings can serve as a theoretical foundation for the further development and implementation of MOLP.

## Materials and methods

### Reagents and materials

*M. oleifera* leaves were purchased from Yunnan Ruziniu Biotechnology (Yunnan, China). The plant material was identified by Prof. Jingui Li. Dextran sulfate sodium (DSS; product code # 160110; MW: 36000–50,000) was obtained from MP Biomedicals (Solon, USA). Myeloperoxidase (MPO) (Cat No. A044-1-1) was obtained from the Jiancheng Bioengineering Institute of Nanjing (Nanjing, China). LPS (Cat No. 21100201) was obtained from the Xiamen Bioendo Technology Co., Ltd. Primers and a bicinchoninic acid (BCA) protein assay kit were provided by Solarbio, Beijing, China. Immobilon-P polyvinylidene fluoride (PVDF) membranes (size: 0.45 μm) were obtained from Merck Millipore (Billerica, USA). RNA-easy Isolation Reagent (Cat No. R701) was purchased from Vazyme Biotech Co., Ltd. Hifair^®^ 1st Strand cDNA Synthesis SuperMix for qPCR (gDNA digester plus) (Cat No. 11141ES60) and Hieff^®^ qPCR SYBR Green Master Mix (High Rox Plus) (Cat No. 11201ES08) were the products of Yeasen Biotech Co., Ltd. Antibodies of TLR4(Cat No. 14358s), MyD88 (Cat No. 4283s), phospho-IκBα (P-IκBα) (Cat No. 4812s), phospho-p65 (P-p65) (Cat No. 8242s) and β-actin (Cat No. 4970s) were the products of Cell Signaling Technology Pathways. TLR4 inhibitor (TAK242, Cat No.M4838) was purchased from Abmole (Houston, TX, USA). Primary antibodies against Occludin and claudin-1 (ab242370) were purchased from Abcam (Cambridge, United Kingdom). The corresponding horseradish peroxidase (HRP)-conjugated secondary antibodies (111–035–003 and 115–035–003) were bought from Jackson Immuno Research (West Grove, PA, United States). Secondary antibody which conjugated tofluorescence (ab150077 and ab150116) was bought from Abcam (Cambridge, United Kingdom). ELISA kits for TNF-α (Cat No. ck-E20852), IL-1β (Cat No. ck-E20174), IL-10 (Cat No. ck-E20162), and HMGB1 (Cat No. ck-E20318) were purchased from Shanghai Beyotime Biotechnology Co., Ltd. (Shanghai, China).

### Preparation and extraction of polysaccharide from *Moringa oleifera*

The crude polysaccharide was extracted from the leaf powder of *M. oleifera* using the procedures reported in earlier investigations (13). The polysaccharide was extracted three times using deionized water at a 1:10 (w/v) ratio at 70°C for 90 min, followed by centrifugation at 4,000 rpm for 20 min. Mixing and evaporating the collected supernatants with a rotary evaporator. Following an overnight incubation at 4°C, then the concentrations were precipitated by adding dehydrated ethanol to a final concentration of 80% (v/v). The obtained precipitates were washed with 95% ethanol and dissolved in deionized water following centrifugation. The dialysate solution was freeze-dried, then deproteinated using the sevage method (14). Final solution was freeze-dried to obtain MOLP.

### Determination of molecular weight

The weight average molecular weight (Mw) of MOLP was determined using SEC-MALLS-RI, which was described in a previous study (15). A DAWN HELEOS-II laser photometer (Wyatt Technology Co., USA) equipped with three tandem columns (300 8 mm, Shodex OH-pak SB-805, 804 and 803; Showa Denko K.K., Tokyo, Japan) was utilized for the determination. MOLP solution (1 mg⋅mL^–1^) filtered through a filter of 0.45 μm pore size, which was held at 45°Cusing a model column heater by Sanshu Biotech. Co., Ltd. (Shanghai, China) and flow rate 0.4 mL⋅min^–1^ with 0.1 M NaNO_3_ aqueous solution containing 0.02% NaN_3_. The Mw was calculated by reference to the standard curve of a Dextran series.

### Monosaccharide composition analysis

5 mg of sample was hydrolyzed with trifluoroacetic acid (TFA, 2 M) at 121°C for 2 h in a sealed tube. The sample was dried with nitrogen. Add methanol to wash, then blow dry, repeat methanol wash 2–3 times. The monosaccharide standards included fucose, arabinose, rhamnose, galactose, glucose, xylose, mannose, fructose, ribose, galacturonic acid and glucuronic acid. Finally, samples were analyzed by high-performance anion-exchange chromatography (HPAEC) on a CarboPac PA-20 anion-exchange column (3 by 150 mm; Dionex) using a pulsed amperometric detector (PAD; Dionex ICS 5000 system). Flow rate, 0.5 mL/min; injection volume, 5 μL; solvent system A: (ddH2O), solvent system B: (0.1 M NaOH), solvent system C: (0.1 M NaOH, 0.2 M NaAc); gradient program, volume ratio of solution A, B, C was 95:5:0 at 0 min, 85:5:10 at 26 min, 85:5:10 at 42 min, 60:0:40 at 42.1 min, 60:40:0 at 52 min, 95:5:0 at 52.1 min, 95:5:0 at 60 min. Data were acquired on the ICS5000 (Thermo Fisher Scientific), and processed using chromeleon 7.2 CDS (Thermo Fisher Scientific).

### Fourier transform-infrared spectroscopy

3 mg of MOLP sample was combined with KBr powder (100 mg) and pressed into thin slices, which were then evaluated in a wave number range of 4,000–400 cm^–1^ using an Fourier transform-infrared (FT-IR) spectrometer (Cary 670-IR + 610-IR, Agilent Company, USA).

### X-Ray diffraction

Five milligram of MOLP sample was measured at an angle of 0.8–140° using an X-ray diffractometer (D8 Advance, Bruker AXS, Germany).

### Scanning electron microscope

MOLP sample was coated with gold powder, and its structural characteristics were analyzed using a scanning electron microscope (SEM) system (S-4800, Hitachi, Japan).

### Animals and experimental design

**FIGURE 1:**
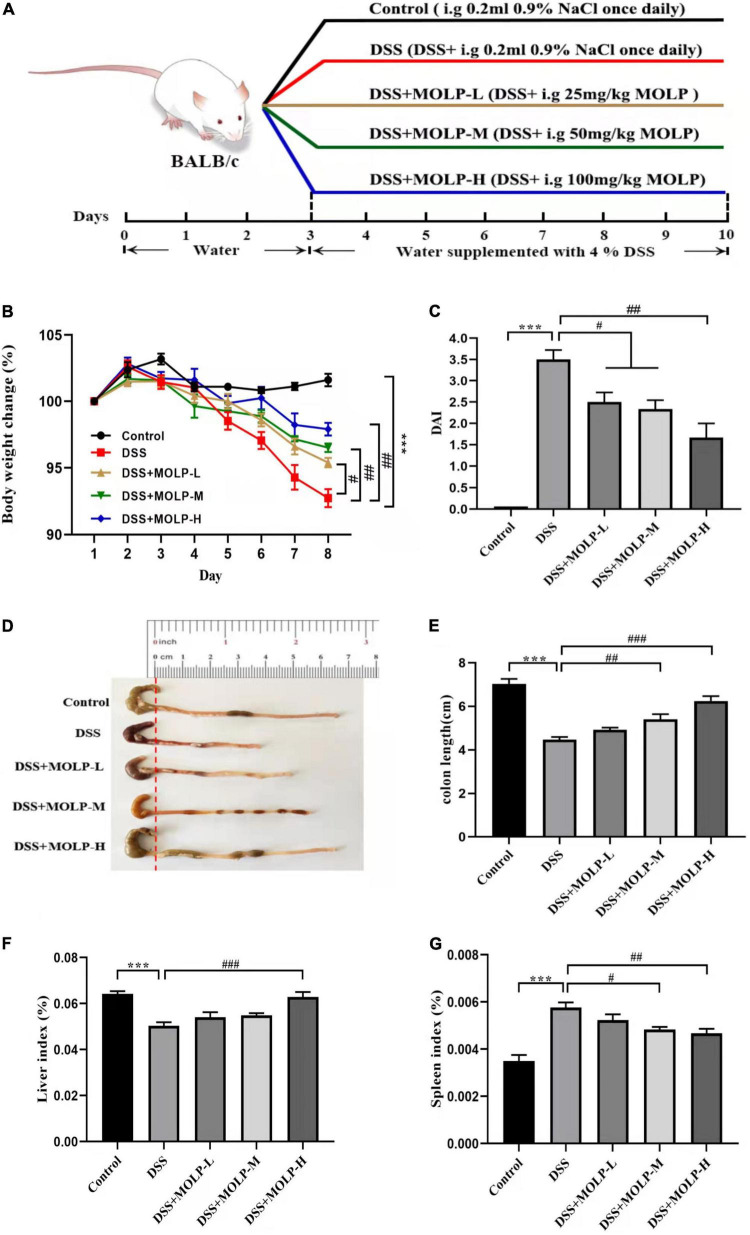
Effect of MOLP on the symptoms of mice with ulcerative colitis. The experimental design **(A)**, changes of body weight **(B)**, DAI **(C)**, the status **(D)** and length **(E)** of colon, indexes of liver **(F)** and spleen **(G)**. Data are presented as mean ± SEM (*n* = 8), ***P < 0.001, DSS vs. Control; ^#^*P* < 0.05, ^##^*P* < 0.01, ^###^*P* < 0.001, DSS + MOLP-L, DSS + MOLP-M and DSS + MOLP-H vs. DSS.

Male BALB/C mice (6–8 weeks old), weighing 20 ± 2 g, were obtained from Yangzhou University Laboratory Animal Co., Ltd. (Yangzhou, China). Forty mice were housed under standard laboratory conditions (12 h light-dark cycle, 25 ± 2°C and 60–80% relative humidity), and fed standard laboratory chow and sterile, distilled water *ad libitum* in the animal room. All animal study was reviewed and approved by Institutional Animal Care and Use Committees (IACUC) of Yangzhou University. After 1 week of acclimatization, the mice were randomly categorized into five groups (*n* = 8). The experimental design was illustrated in Figure 1A. All experimental groups were administered distilled water for the first 3 days, control group was administered 0.9% (0.2 mL) sodium chloride (NaCl) from days 4 to 10. DSS group was administered 4% (w/v) DSS from days 4 to 10. DSS + MOLP-L group, DSS + MOLP-M group and DSS + MOLP-H group were given oral administration with different doses of MOLP (25, 50 and 100 mg/kg/day, respectively), along with the oral administration of 4% (w/v) DSS from days 4 to 10 for 7 days. All mice were injected with 0.1% (50 mg/kg, i. p.) pentobarbital sodium and sacrificed after 10-day experimental period. The weight of liver and spleen were measured and recorded. The entire colon was quickly removed and washed with cold phosphate-buffered saline (PBS). The distal part was fixed in 10% buffered formalin for histological analysis, and other parts were then stored at –80°C for immunological assays.

### Disease activity index and histological injury analysis

The body weight of mice was recorded using an electronic analytical balance. The following analysis of a disease activity index (DAI) indicator score, which included body weight loss, stool consistency, and blood in the stools, was performed in accordance with the literature (16). Briefly, (a) body weight loss: 0 points = none; 1 points = 1–5% loss; 2 points = 5–10% loss; 3 points = 10–20% loss; 4 points = over 20% loss. (b) Diarrhea: 0 points = normal; 1 point = soft but still formed; 2 points = soft; 3 points = very soft and wet; 4 points = watery diarrhea. (c) Hematochezia: 0 points = negative hemoccult; 1 point = weakly positive hemoccult; 2 points = positive hemoccult; 3 points = blood traces in stool visible; 4 points = gross rectal bleeding.

Using the previously published method (17), paraffin-embedded colonic tissues were sectioned and stained with hematoxylin and eosin (H&E) for evaluation and histopathologic scoring of UC. Briefly, (a) severity of inflammation: 0 points = none; 1 points = mild; 2 points = moderate; 3 points = severe. (b) Extent of inflammation: 0 points = none; 1 points = mucosal; 2 points = mucosal and submucosal; 3 points = transmural. (c) Crypt damage: 0 points = none; 1 points = basal 1/3; 2 points = basal 2/3; 3 points = crypts lost but surface epithelium present; 4 points = crypts and surface epithelium lost.

### Determination of myeloperoxidase activity, inflammatory cytokines and lipopolysaccharide content

Myeloperoxidase (MPO) activity, and inflammatory cytokines such as TNF-α, IL-1β, HMGB1 and IL-10 levels in colonic tissues, and serum lipopolysaccharide (LPS) levels were assessed using ELISA kits in accordance with the manufactures instructions.

### Analysis of colon tissues after dextran sulfate sodium-treatment using qRT-PCR

Using Trizol reagent, total RNA was isolated from colon tissue samples and examined using a NanoDrop 2000 UV-vis spectrophotometer (Thermo Fisher Scientific, Wilmington, DE, USA). The Hifair^®^ I1st strand cDNA synthesis SuperMix was used to create the cDNA samples. Utilizing the CFX96™ connect real-time PCR system (Bio-Rad, USA) and SYBR^®^ Green Master Mix Kit, the expression levels of IL-1β, IL-10, TNF-α, HMGB1 and PPAR-mRNA in colon tissues were determined. GAPDH was designated as the housekeeping gene for the 2^–Δ^ ^Δ^ ^CT^ technique to calculate relative mRNA levels. The specific primers for the target genes are shown in Table 1.

**TABLE 1:** List of primer sequences used for qRT-PCR.

Gene	Sense (5′–3′)	Antisense (5′–3′)
IL-1β	CCAGCAGGTTATCATCACATCC	ATCTCGCAGCAGCACATCA
IL-10	GGCAGCCTTGTCCCTTG	AACATACTGCTAACCGACTCCTT
TNF-α	TGAAGCAGCAGCCAGCAA	GCAGCCTGTCTCCTTCTATGA
HMGB1	ATGGGCAAAGGAGATCCTA	ATTCATCATCATCATCTTCT
PPARγ	CCCACCAACTTCGGAATCAG	TGCTGGAGAAATCAACCGTGGTA
GAPDH	CACCATCTTCCAGGAGCGAG	GGGGCCATCCACAGTCTTC

### Western blotting analysis

The colon tissue was homogenized and lysed in RIPA buffer with a PMSF protease inhibitor on ice, homogenized, and centrifuged at 12,000 rpm for 10 min. The amount of proteins in the supernatant was then measured using a BCA protein assay kit. After boiling with loading buffer, equal quantities of protein from each sample were prepared for electrophoresis on 10% SDS-PAGE under reducing conditions, transferred to the PVDF membrane, blocked for 2 h with 5% skim milk, and then overnight incubated with primary antibodies (1:1,000 dilution) at 4°C (all antibodies were diluted following instructions). The membrane was treated with species-specific secondary antibodies together with horseradish peroxidase (1:1,000 dilution) at room temperature for 1 h after three TBST washes. Using an enhanced chemiluminescence kit, protein signal bands were observed on a Chemidoc XRS (BIO-RAD, Marnes-la-Coquette, France) following three washes with TBST (Merck Millipore, Billerica, USA). and quantified using Image J software.

### Immunohistochemistry analysis

Paraffin-embedded slices of colonic tissue were deparaffinized, rehydrated, rinsed with distilled water, and then placed in citrate buffer for antigen thermal retrieval. After that, the cells were washed three times with PBS for 5 min each, incubated for 60 min at room temperature with blocking buffer (3% BSA in PBS), and then incubated overnight at 4°C with anti-zonula occludens-1 (ZO-1) and anti-occludin primary antibodies. The primary antibody was incubated with the secondary antibody for 50 min at room temperature before being rinsed three times with PBS for 5 min each and stained with DAPI for 10 min. After being acquired with a fluorescent microscopy imaging system (Nikon Corporation, Tokyo, Japan), the sections were quantified using Image J software.

### Statistical analysis

The data is displayed as the mean ± standard error of the mean. GraphPad Prism’s one-way ANOVA test was utilized for statistical analysis (version 8.0). *P* < 0.05, *P* < 0.01, or *P* < 0.001 indicates statistical significance.

## Results and discussion

### Molecular weight and monosaccharide composition

**FIGURE 2:**
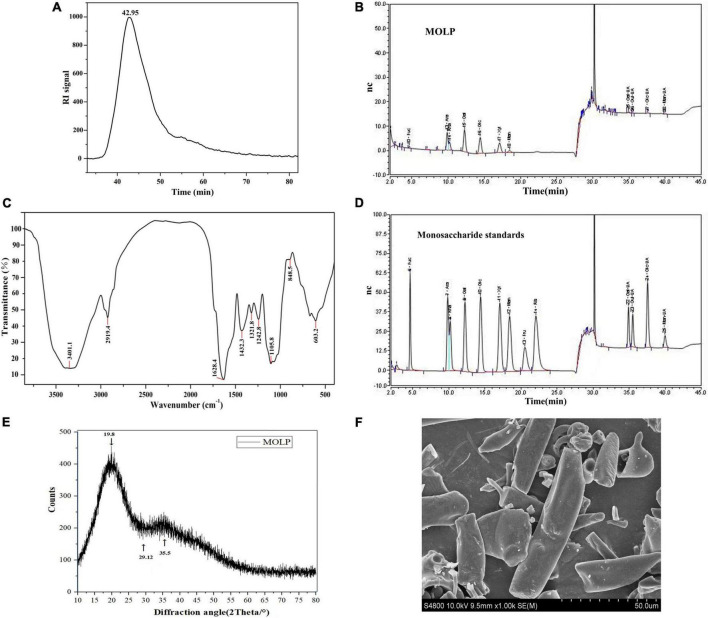
Structural characterization of MOLP. The peak at retention time of MOLP **(A)**. The monosaccharide composition analysis of MOLP **(B)**. FT-IR spectrum of MOLP **(C)**. Monosaccharide standards **(D)**. Fuc, fucose; Ara, arabinose; Rha, rhamnose; Gal, galactose; Glc, glucose; Xyl, xylose; Man, mannose; Gal-UA, galacturonic acid; Glu-UA, glucuronic acid; Man-UA, mannose uronic acid. XRD **(E)**, and SEM **(F)** of MOLP.

The Mw distribution of MOLP was determined by SEC-MALLS-RI. As shown in Figure 2A, a single, sharp, and symmetrical peak at 42.95 min was observed, indicating that MOLP was a homogeneous polysaccharide. The Mw of MOLP was estimated to be 182,989 kDa.

The monosaccharide composition analysis of MOLP was shown in Figure 2B, MOLP was composed of fucose, arabinose, rhamnose, galactose, glucose, xylose, mannose, galactose uronic acid, glucuronic acid, glucose uronic acid and mannose uronic acid, with a percentage ratio of 1.64, 18.81, 12.04, 25.90, 17.57, 12.01, 3.51, 5.28, 0.55, 1.27, and 1.43%, respectively. According to the standard curve (Figure 2D), the result indicated that MOLP was a hetero-polysaccharide.

### Fourier-transform infrared spectroscopy analysis

The FT-IR spectrum of MOLP revealed typical polysaccharide absorption peaks in the ranges of 4,000–400 cm^–1^. As shown in Figure 2C, the strong absorptions at 3401.1 and 2919.4 cm^–1^ showed O–H and C–H stretching vibrations, respectively (18). The absorption peak at 1628.4 cm^–1^ was given the C = O stretching vibration. The absorption peak at 1242.8 cm^–1^ was identified as the source of the S = O stretching vibration. The absorption peak at 1105.8 cm**^–^**^1^ was also attributed to the C–O stretching vibration. Additionally, a pyranose ring was discovered by the 848.5 cm^–1^ absorption, it is similar with the previous studies on the polysaccharides extracted from the leaves of *M. oleifera* (19, 20).

### X-ray diffraction analysis

An obvious dispersing peak was observed at 19.80° according to the XRD curve of MOLP (Figure 2E). However, no obvious characteristic peaks were observed, only a few small dispersing absorption peaks at 29.12° and 35.50°, respectively.

### Scanning electron microscope analysis

The SEM image of MOLP was illustrated in Figure 2F. The surface of MOLP was smooth and mainly exhibited sheet and needle or rod-like shape.

### *Moringa oleifera* leaves polysaccharide improved colitis symptoms in dextran sulfate sodium-treated mice

The change in body weight was calculated and shown in Figure 1B. The DSS treatment led to a significant weight loss. Nonetheless, MOLP administration had a protective effect and reduced the trend in body weight loss in a dose-dependent manner, indicating that MOLP reduced body weight loss in DSS-induced UC mice.

DAI scores were used to detect the progression of DSS-induced UC (21). Colonic contraction has been identified as the primary feature of UC, and shortening of colon length is clearly associated with disease severity (22). As illustrated in Figure 1C, DSS treatment significantly increased DAI score in model mice after 10 days. However, MOLP supplementation inhibited the elevation of DAI scores in the low, medium, and high dose groups. In contrast, the DSS group showed a significant contraction in mouse colon length. Nonetheless, intervention with MOLP at medium and high doses effectively prevented colon shortening (Figures 1D,E). Similar to Zhang et al. (23), which indicated that 5-ASA and (MOPE) isolated from *M. oleifera* treatments reduced inflammatory symptoms in the colon of mice. Our results suggested that the MOLP supplementation has a preventative effect on UC induced by DSS.

Spleen index is regarded as a key indicator of immunological function (24). In this study, DSS challenge significantly reduced the liver index while increasing the spleen index when compared to the control. However, the high dose of MOLP significantly increased the liver index while decreasing the spleen index in DSS-induced UC mice (Figures 1F,G). Taken together, all the findings showed that MOLP reduced clinical symptoms and anatomical changes in DSS-induced UC.

### *Moringa oleifera* leaves polysaccharide ameliorated the colonic histopathological changes

**FIGURE 3:**
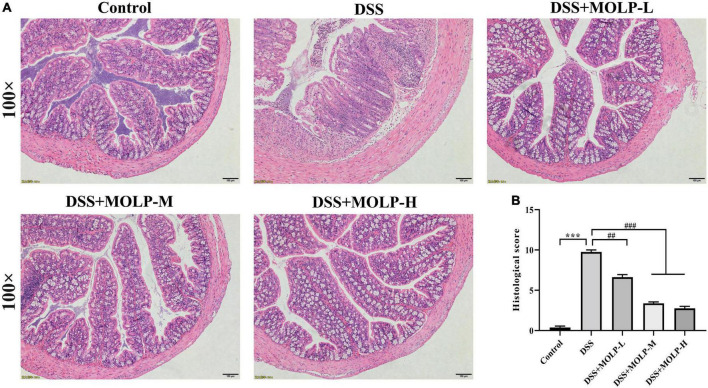
Effect of MOLP on histopathological changes of mice with ulcerative colitis. Histology analysis of colon tissues by HE staining **(A)**, histological sore **(B)**. Data are presented as mean ± SEM (*n* = 8), ***P < 0.001, DSS vs. Control; ^##^*P* < 0.01, ^###^*P* < 0.001, DSS + MOLP-L, DSS + MOLP-M and DSS + MOLP-H vs. DSS.

As shown in Figure 3, the histology of the control group was normal, with dense columnar epithelium, intact intestinal crypts, and abundant goblet cells. There was epithelial rupture, irregular crypt architecture, submucosal edema, goblet cell reduction, and neutrophil hyper-infiltration in the DSS group. On the contrary, MOLP intervention significantly improved these DSS-induced histopathological scores. According to previous studies, the impaired function of the intestinal mucosal barrier is directly related to the development of UC (25) and was characterized by epithelial rupture, irregular mucosal and crypt structure, and reduction of goblet cells. And also neutrophils are one of the infiltrating cells that cause inflammation in colitis (26). This is consistent with the findings of Hong et al. (27), which indicated that a high dose of (MOP) extracted and purified a peptide from *M. oleifera* seeds significantly reduced such mucosal damage (including the greater crypt depth), decreased in goblet cells and inflammatory cell infiltration, leading to lower histological scores in DSS-induced UC mice. Therefore, these results showed that MOLP supplementation could improve the histopathological changes in DSS-induced UC.

### *Moringa oleifera* leaves polysaccharide decreased myeloperoxidase activity in colonic tissues and serum lipopolysaccharide level

MPO is a peroxidase that reflects infiltration and inflammation levels directly (28). LPS is another inflammatory stimulator that promotes the release of pro-inflammatory cytokines, inflammatory signaling, and tissue damage in a range of inflammatory diseases (29). We examined MPO activity in colon tissues and serum LPS levels in DSS-induced UC mice. As shown in Table 2, MPO activity and LPS level raised significantly in the DSS treatment relative to the control group. Additionally, administration reduced tissue MPO activity and serum LPS levels in the DSS + MOLP-M and DSS + MOLP-H groups when compared to the DSS group. This is in line with the results of Hong et al. (27), which indicated that a high dose of (MOP) decreased MPO in the serum of DSS-induced UC mice. These findings suggest that MOLP plays a protective role against DSS-induced UC by inhibiting MPO activity and serum LPS levels.

**TABLE 2:** Effects of MOLP on MPO activity and LPS content.

Groups	Control	DSS	DSS + MOLP-L	DSS + MOLP-M	DSS + MOLP-H
MPO (U/g)	0.11 ± 0.03	0.54 ± 0.07**	0.33 ± 0.03	0.29 ± 0.09^#^	0.15 ± 0.08^##^
LPS (EU/mL)	1.61 ± 0.06	4.87 ± 0.57***	3.71 ± 0.18	2.53 ± 0.07^##^	2.37 ± 0.09^##^

Data are presented as mean ± SEM (*n* = 8), **P < 0.01, ***P < 0.001, DSS vs. Control; ^#^*P* < 0.05, ^##^*P* < 0.01, DSS + MOLP-M and DSS + MOLP-H vs. DSS. MPO, Myeloperoxidase; LPS, Lipopolysaccharide; DSS, Dextran sodium sulfate; MOLP-L, low dose of *Moringa oleifera* leaf polysaccharide; MOLP-M, medium dose of *Moringa oleifera* leaf polysaccharide; MOLP-H, high dose of *Moringa oleifera* leaf polysaccharide.

### Anti-inflammatory effect of *Moringa oleifera* leaves polysaccharide in the colon tissues

**FIGURE 4:**
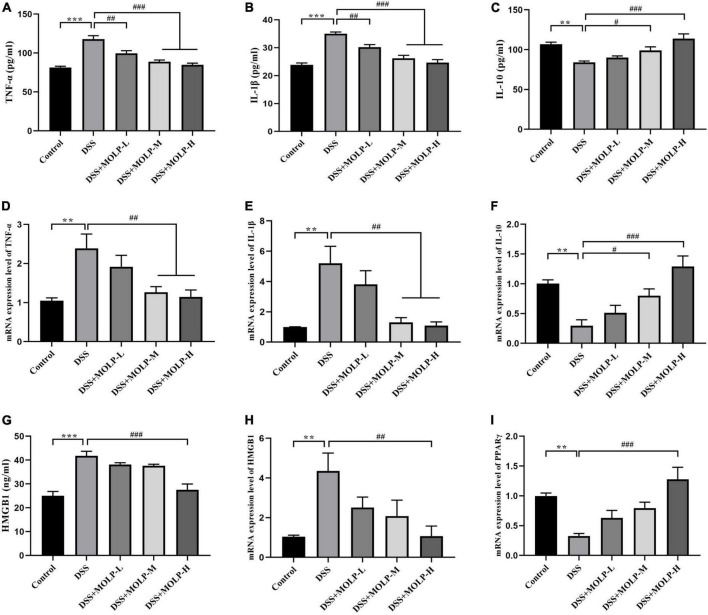
Effect of MOLP on serum cytokine levels and colon tissues in mice with ulcerative colitis. The concentrations of TNF-α **(A)**, IL-1β **(B)**, IL-10 **(C)** and HMGB1 **(G)** detected by (ELISA) (*n* = 8). The mRNA expression levels of TNF-α **(D)**, IL-1β **(E)**, IL-10 **(F)**, HMGB1 **(H)** and PPARγ **(I)** (*n* = 5). Data are presented as mean ± SEM, **P < 0.01, ***P < 0.001, DSS vs. Control; ^#^*P* < 0.05, ^##^*P* < 0.01, ^###^*P* < 0.001, DSS + MOLP-L, DSS + MOLP-M and DSS + MOLP-H vs. DSS.

TNF-α is a major factor promoting damage to the intestinal epithelial barrier, is associated with the development of UC, and has the potential to stimulate the production of IL-1β (30, 31). The anti-inflammatory cytokine IL-10 can inhibit the production of pro-inflammatory cytokines such as TNF-α and IL-1β (4). In addition, HMGB1 is a key mediator in the pathogenesis of systemic inflammation in a variety of inflammatory diseases, with a strong ability to trigger inflammatory responses (32). In order to determine whether MOLP intervention might alleviate DSS-induced colonic inflammation by modulating the inflammatory response, the levels of inflammatory cytokines and their mRNA in colon tissues were measured. The expression levels of pro-inflammatory cytokines TNF-α, IL-1β and HMGB1 were up-regulated after oral administration of DSS compared with the control group (Figure 4). However, the expression levels of TNF-α, and IL-1β were dramatically reduced in the colon tissue of colitis mice after MOLP intervention at all doses (Figures 4A,B). While, the IL-10 expression level in colon tissues was up-regulated after the medium and high doses of MOLP treatment (Figure 4C). Only at the high dose of MOLP intervention was the level of HMGB1 significantly lower than in the DSS group (Figure 4G).

Similar trends were shown in terms of mRNA expression levels of inflammatory cytokines in colon tissues. The mRNA expression levels of TNF-α, IL-1β and HMGB1 were significantly up-regulated by DSS challenge. In contrast, the mRNA expressions levels of TNF-α and IL-1βwere significantly down-regulated by MOLP administration at the medium and high dosages (Figures 4D,E). And the level of HMGB1 mRNA expression in the DSS + MOLP-H group was significantly lower than that in the DSS group (Figure 4H). However, the IL-10 mRNA expression levels were significantly increased in the DSS + MOLP-M and DSS + MOLP-H groups (Figure 4F). Previous studies reported that polysaccharides play important roles in cytokine homeostasis by regulating inflammatory factor levels (33, 34).

PPAR-γ is highly expressed in intestinal and colonic mucosal epithelial cells, as well as macrophages (35). The level of PPAR-γ mRNA expression in colon tissue was investigated. As showed in Figure 4I, DSS treatment alone significantly down-regulated the PPAR-γ mRNA expression level. However, the DSS-induced change in mice may incrementally revert to normal level by the high dose of MOLP administration. Previous studies reported that mRNA PPAR-γ expression were decreased in active UC compared to the UC in remission (36), and also its expression were significantly lower in comparison to healthy controls (37). According to Li et al. (13) MOP supplementation effectively suppressed serum concentration levels of TNF-a and IL-1β, and regulated the mRNA expression level of PPARγ in high-fat diet (HFD)-induced C57BL/6J mice. Our findings suggested that MOLP supplementation regulated inflammatory responses in DSS-induced UC by suppressing anti-inflammatory cascades.

### *Moringa oleifera* leaves polysaccharide inhibited the TLR4/MyD88/NF-κB signaling pathway in colonic tissues

**FIGURE 5:**
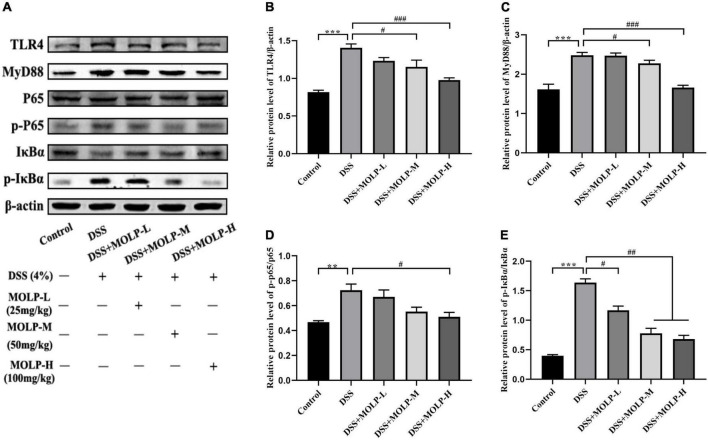
Effect of MOLP on colon tissues in mice with ulcerative colitis. Western blot analysis of key signaling proteins in colonic tissue **(A)**, TLR4 **(B)**, MyD88 **(C)**, NF-κB p65/p-P65 **(D)** and p-IκBα/IκBα **(E)**. Data are presented as mean ± SEM (*n* = 3), **P < 0.01, ***P < 0.01, DSS vs. Control; ^#^*P* < 0.05, ^##^*P* < 0.01, ^###^*P* < 0.001, DSS + MOLP-L, DSS + MOLP-M and DSS + MOLP-H vs. DSS.

NF-κB signaling pathway is important in the development of UC (38, 39). In comparison to the DSS group, medium and high doses of MOLP supplementation effectively reduced the up-regulation of TLR4 and MyD88 expression in colonic tissues induced by DSS (Figures 5B,C). Oral administration with DSS resulted in a marked increase in IκBα phosphorylation (p-IκBα), IκBα is a key inhibitor of NF-κB activation, and MOLP inhibited p-IκBα (Figure 5E). Furthermore, elevated phosphorylation of NF-κB p65 (p-NF-κB p65) was observed in colon tissue after DSS challenge, whereas high-dose MOLP supplementation significantly blocked DSS-induced p-NF-κB p65 (Figure 5D). Previous studies found that MOPE reduced the protein expression of NF-κB p65 and p-IκBα while increasing the expression of IκBα (23). Therefore, these results suggested that MOLP may suppress inflammatory responses by inhibiting TLR4/MyD88/NF-κB signaling pathways (Figure 5A), thereby reducing DSS-induced UC in mice.

### *Moringa oleifera* leaves polysaccharide attenuated dextran sodium sulfate -induced loss to colonic epithelial tight junction proteins

**FIGURE 6:**
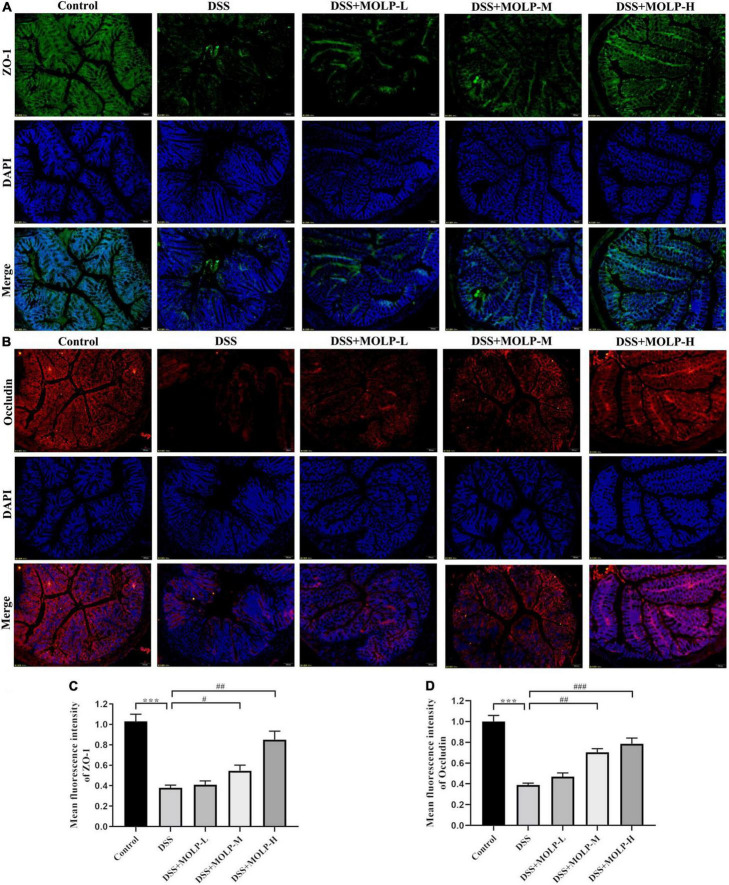
Effect of MOLP on the tight junction of mice with ulcerative colitis. Sections of colon tissues were immunostained with DAPI and antibodies and then observed under 200 × fluorescence microscope. The green fluorescence represents the amount of ZO-1 **(A)**. The red fluorescence represents the amount of occludin **(B)**. The blue fluorescence is the nucleus stained by DAPI. **(C)** quantified results from **(A)**, **(D)** quantified results from **(B)**. Data are presented as mean ± SEM (*n* = 3), ***P < 0.001, DSS vs. Control; ^#^*P* < 0.05, ^##^*P* < 0.01, ^###^*P* < 0.001, DSS + MOLP-M and DSS + MOLP-H vs. DSS.

The intercellular TJ proteins are essential components of the intestinal mechanical barrier and are responsible for epithelial permeability, paracellular spreading, and intercellular adhesion (40, 41). TJs, including ZO-1 and occludin, are essential to maintain intestinal integrity (42). However, natural extracts have been shown in clinical studies to improve the expression of TJ proteins, thereby maintaining the intestinal barrier and preventing the development of UC (43). Previously, alterations in the TJ protein of colonic epithelial cells exacerbate colitis (44). In this study, immunofluorescence staining was used in the colons of mice to detect the distribution and expression of the intracellular scaffold protein ZO-1 and the transmembrane TJ protein occludin to see whether physiological changes in the barrier were related to changes in TJ protein distribution. As showed in the Figure 6, the fluorescence intensity of ZO-1 was significantly attenuated in DSS treated mice, and similar to the changes in the occludin expression. However, the medium and high doses of MOLP administration maintained the expression and distribution of these two proteins. Previous research (27) found that MOP reduced the levels of occludin and ZO-1 in DSS-induced UC mice. These findings indicated that the protective roles of MOLP in the intestinal epithelium may be associated with its ability to improve compromised TJs in DSS-induced UC.

**FIGURE 7:**
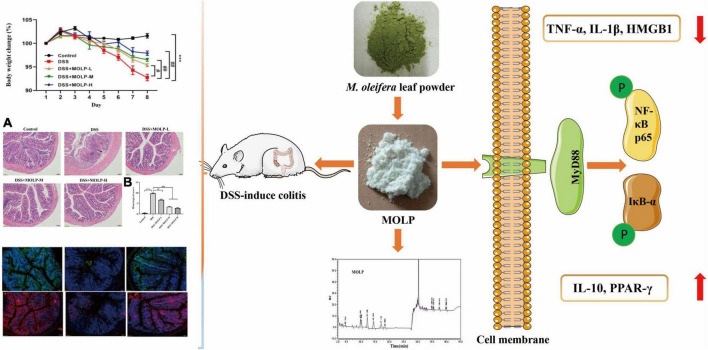
Diagram showing the effect of MOLP on DSS-induced UC. MOLP alleviated colonic pathological alterations, decreased goblet cells, crypt destruction, and infiltration of inflammatory cells caused by DSS **(A)**, and decreased the histological score **(B)**. MOLP repressed the loss of ZO-1 and occludin proteins in mucosal layer. MOLP suppressed TNF-α, IL-1β, and HMGB1 production. MOLP increased the mRNA expression of IL-10 and PPAR-γ, and decreased the activation of MyD88/NF-κB signaling pathway.

Taken together, MOLP alleviated colonic pathological alterations, decreased goblet cells, crypt destruction, and infiltration of inflammatory cells caused by DSS. MOLP suppressed the loss of ZO-1 and occludin proteins in mucosal layer and the production of TNF-α, IL-1β and HMGB1. Furthermore, MOLP increased the mRNA expression of IL-10 and PPAR-γ, and decreased the activation of TLR4, MyD88, NF-κB signaling pathway (Figure 7).

## Conclusion

Our study confirmed that the polysaccharide obtained from *M. oleifera* leaf exhibited prophylactic efficacies on DSS-induced UC by reducing intestinal damage, suppressing the activation of TLR4/MyD88/NF-κB signaling pathway, as well as the release of cytokines that promote inflammation, whereas maintaining the goblet cells and expression of TJ proteins. These findings will make a better understanding of the protective action of MOLP against UC, thereby providing a rationale for the development of MOLP.

## Data availability statement

The original data presented in this study are included in the Supplementary material, further inquiries can be directed to the corresponding authors.

## Ethics statement

This animal study was reviewed and approved by the Institutional Animal Care and Use Committees (IACUC) of Yangzhou University.

## Author contributions

HM: conceptualization, methodology, software, data curation, and writing—original draft. WP: conceptualization, methodology, investigation, software, data curation, writing—original draft, and visualization. HS and RZ: methodology, investigation, and writing—original draft. YT: investigation and software. JH: visualization and investigation. ML: supervision. RB and JL: conceptualization, methodology, project administration, and funding acquisition. All authors contributed to the article and approved the submitted version.

## Abbreviations

UC: ulcerative colitis
IBD: inflammatory bowel disease
MOLP: *Moringa oleifera* leaves polysaccharide
TNF- α: tumor necrosis factor alpha
DSS: dextran sulfate sodium
MPO: myeloperoxidase
TJ: tight junction
DAI: disease activity index
PPAR- γ: peroxisome proliferator-activated receptor- γ
HMGB1: High mobility group box 1
ZO-1: zonula occludens-1
TLR4: Toll-like receptor 4
NF- κ B: nuclear factor-kappa B
I κ B α: inhibitor of kappa B alpha
MyD88: myeloid differentiation primary response 88.
