# Supplementary figures and images for: Moringa oleifera leaf polysaccharide alleviates experimental colitis by inhibiting inflammation and maintaining intestinal barrier
Source: Front Nutr. 2022 Nov 10;9:1055791. doi: 10.3389/fnut.2022.1055791 (PMC9686441; doi:10.3389/fnut.2022.1055791)

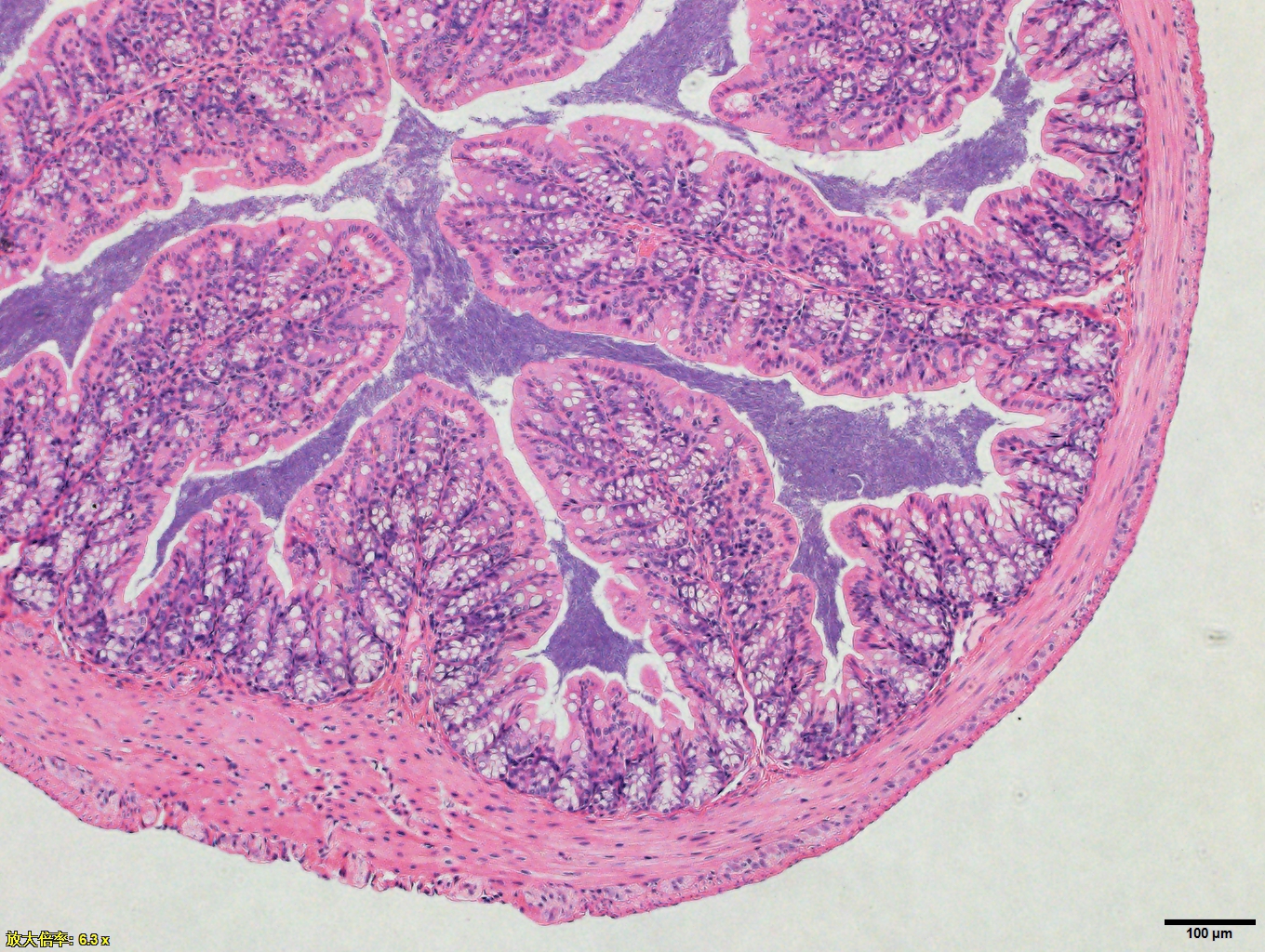

Supplement: Supplementary file 1 [file Data_Sheet_1.zip › Figure 3/Control 100x.jpg]

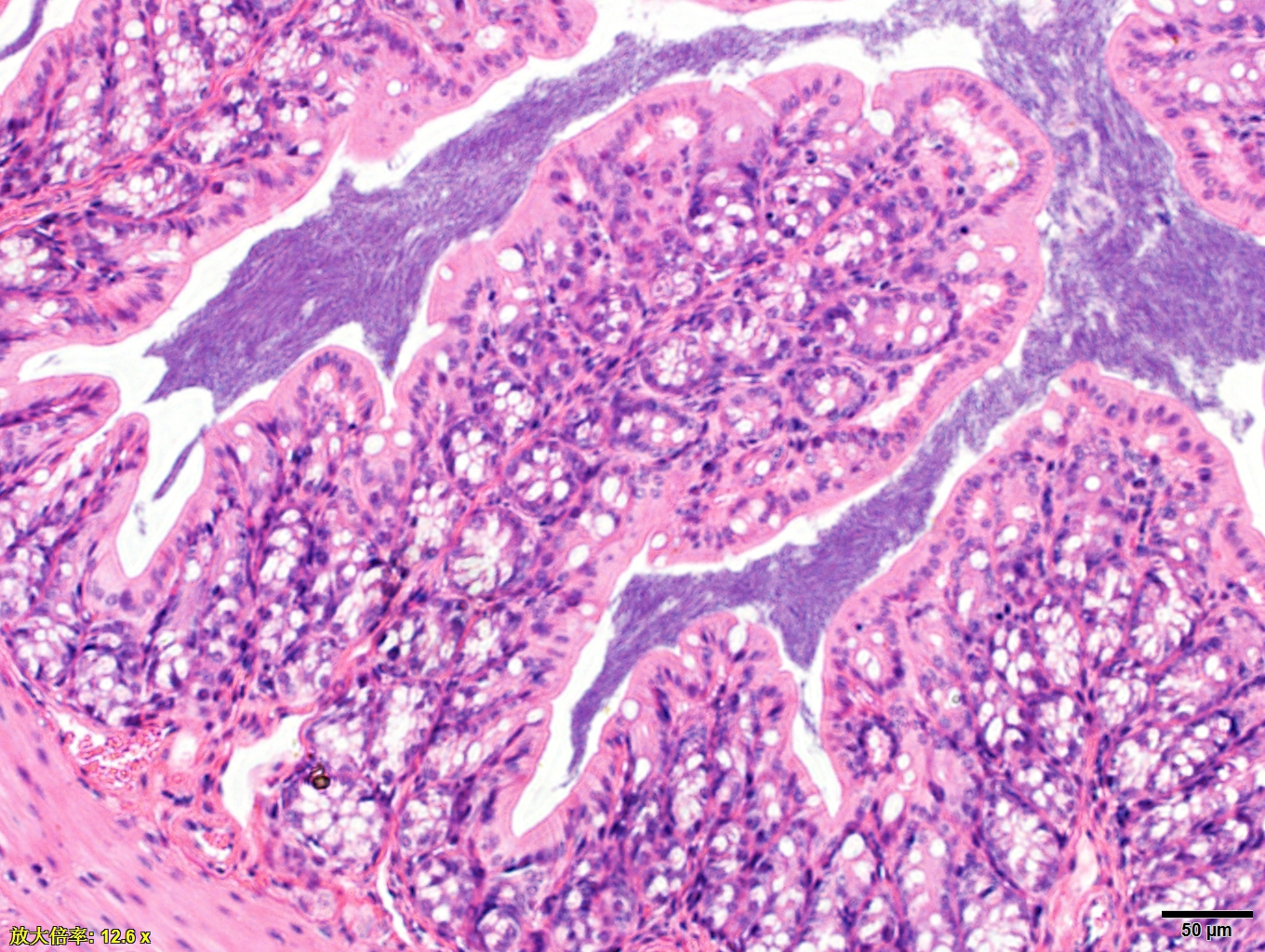

Supplement: Supplementary file 1 [file Data_Sheet_1.zip › Figure 3/Control 200x.jpg]

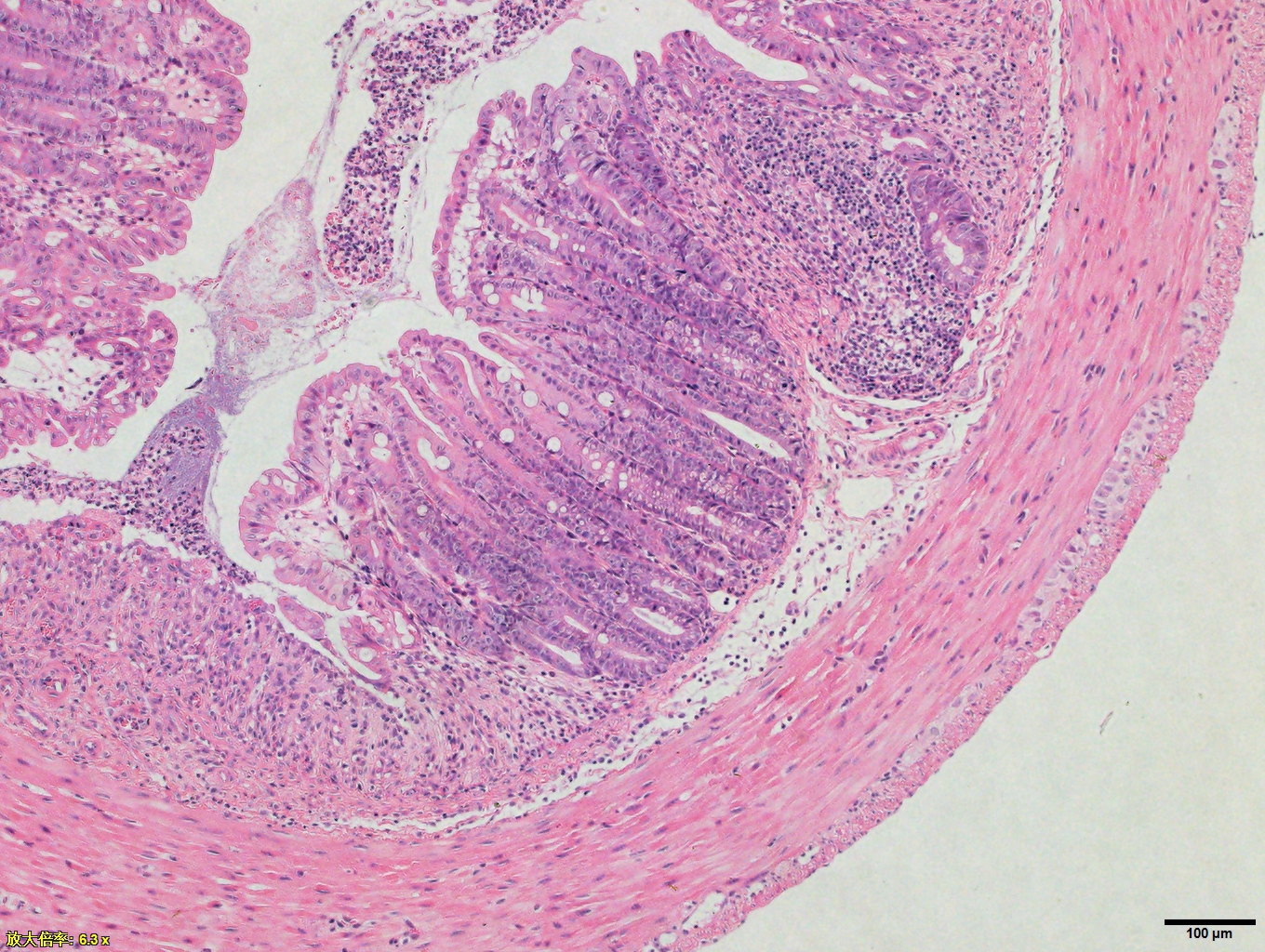

Supplement: Supplementary file 1 [file Data_Sheet_1.zip › Figure 3/DSS 100x.jpg]

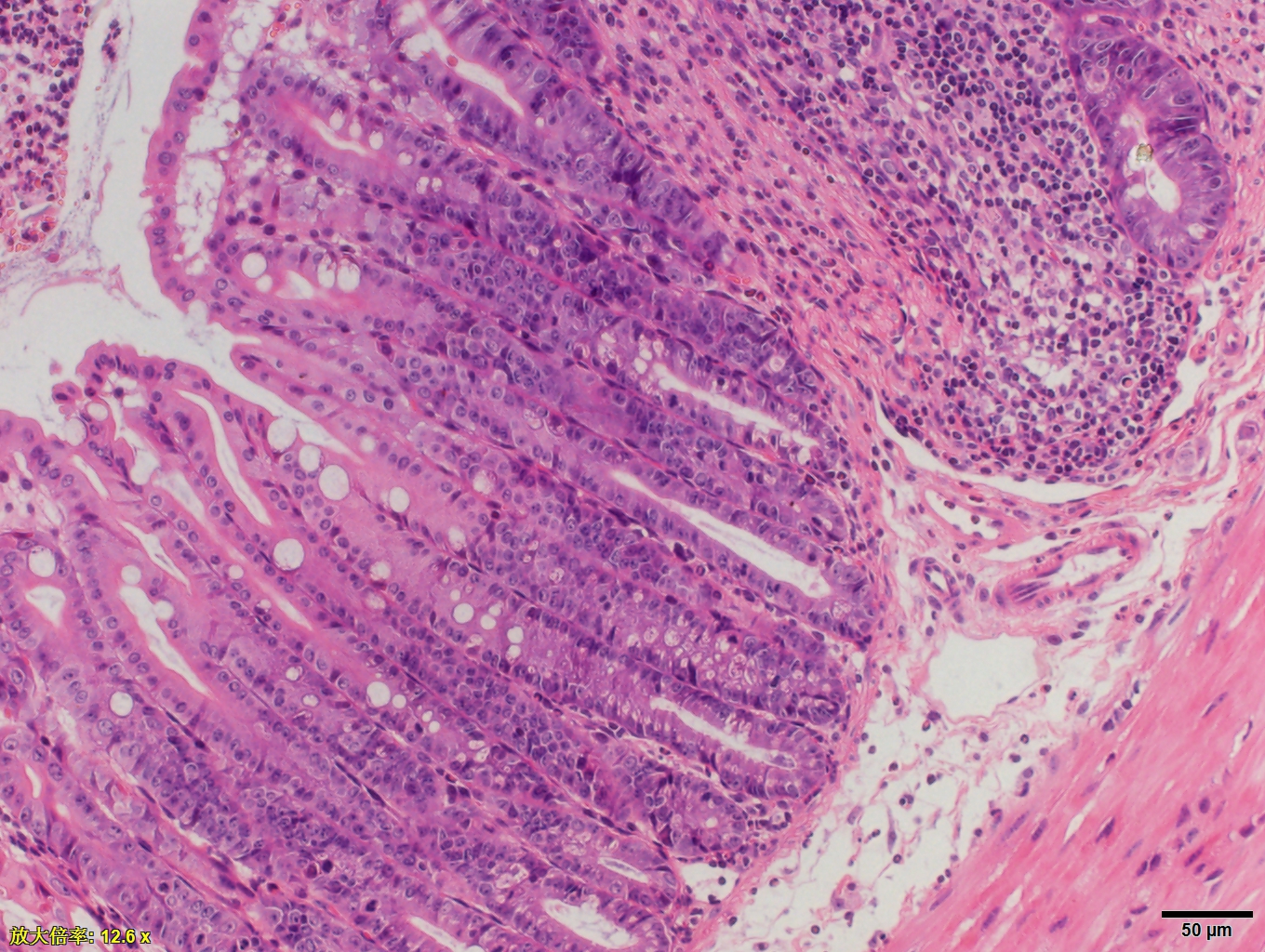

Supplement: Supplementary file 1 [file Data_Sheet_1.zip › Figure 3/DSS 200x.jpg]

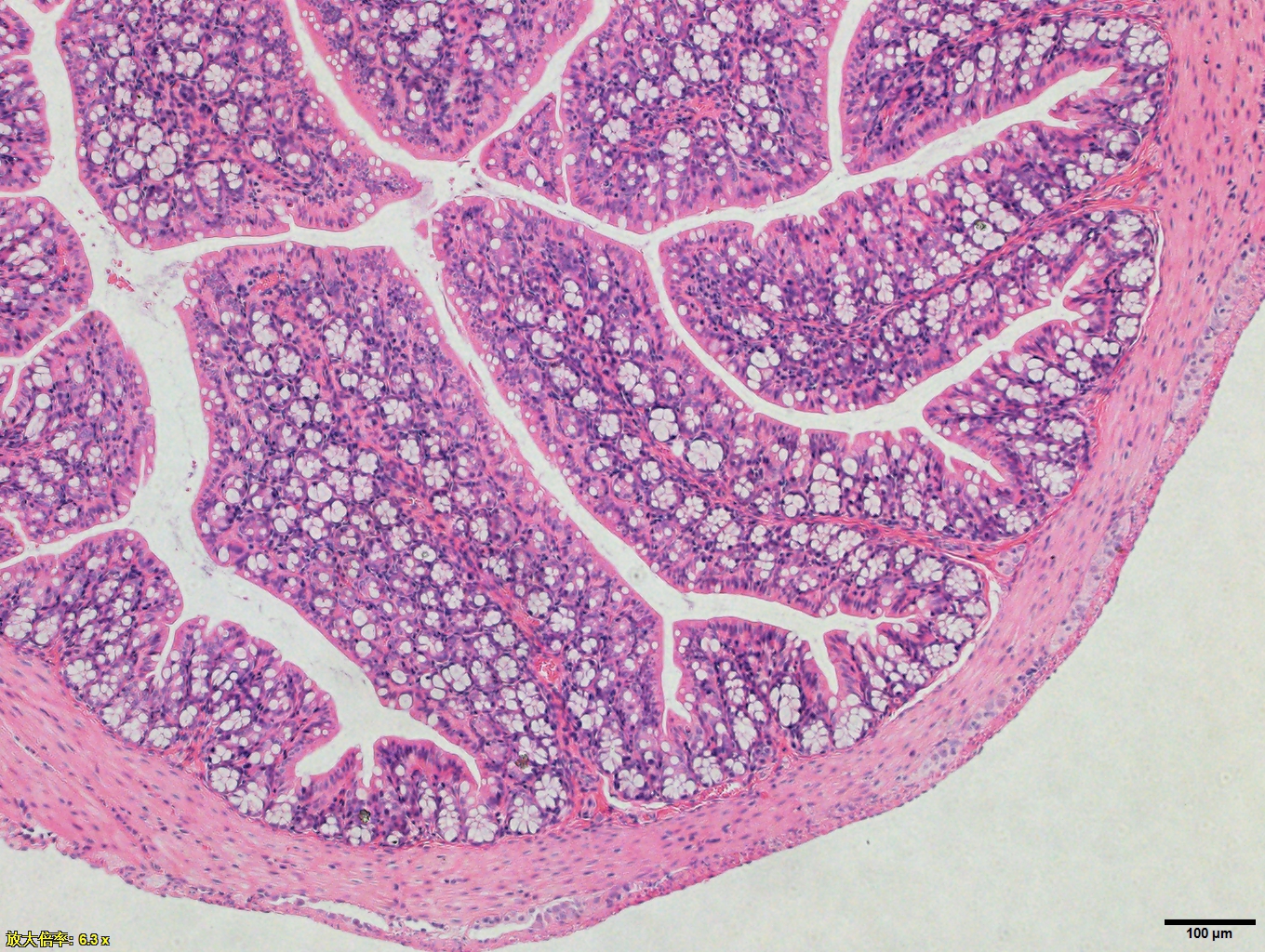

Supplement: Supplementary file 1 [file Data_Sheet_1.zip › Figure 3/DSS+MOLP-H 100x.jpg]

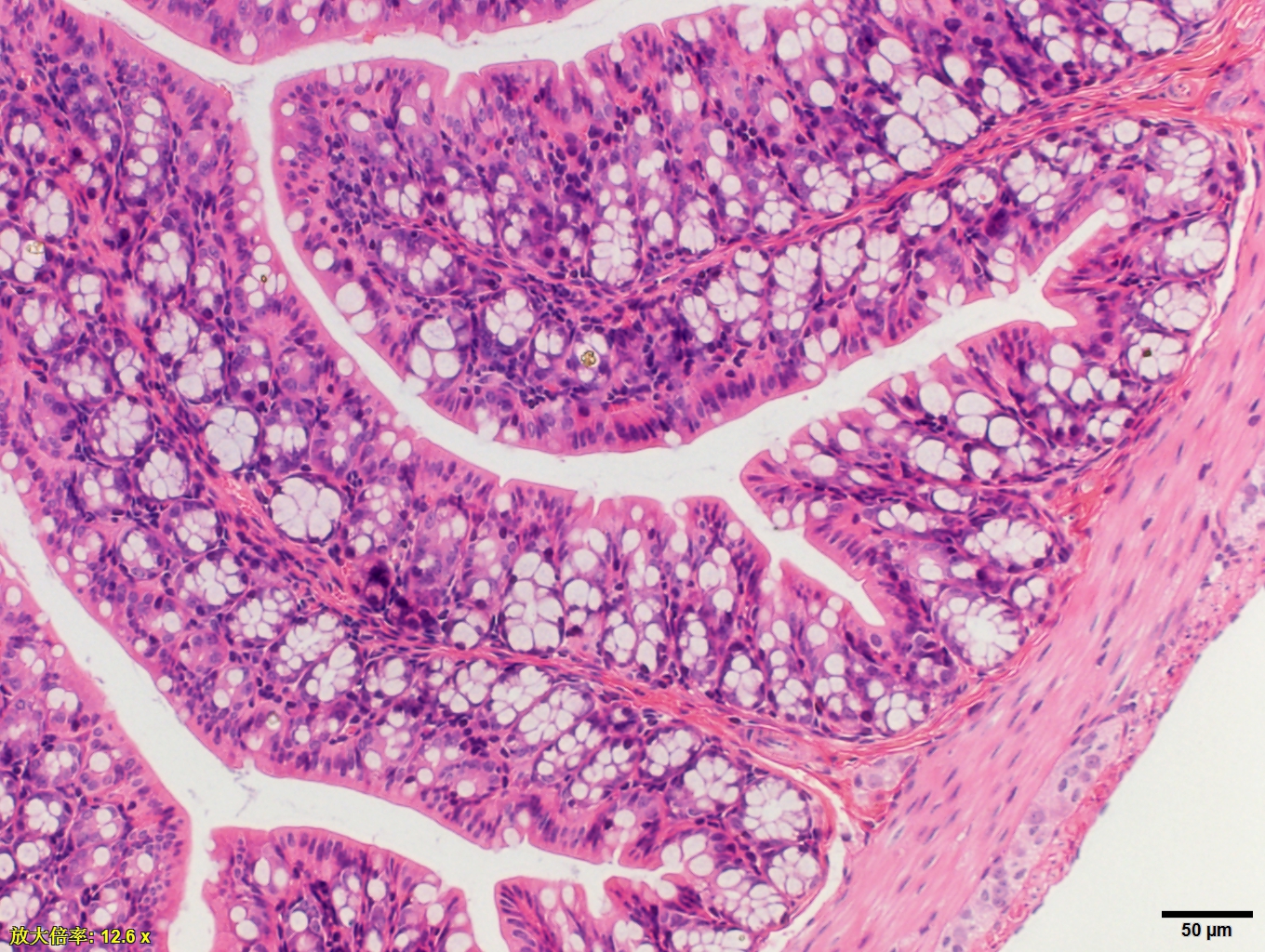

Supplement: Supplementary file 1 [file Data_Sheet_1.zip › Figure 3/DSS+MOLP-H 200×.jpg]

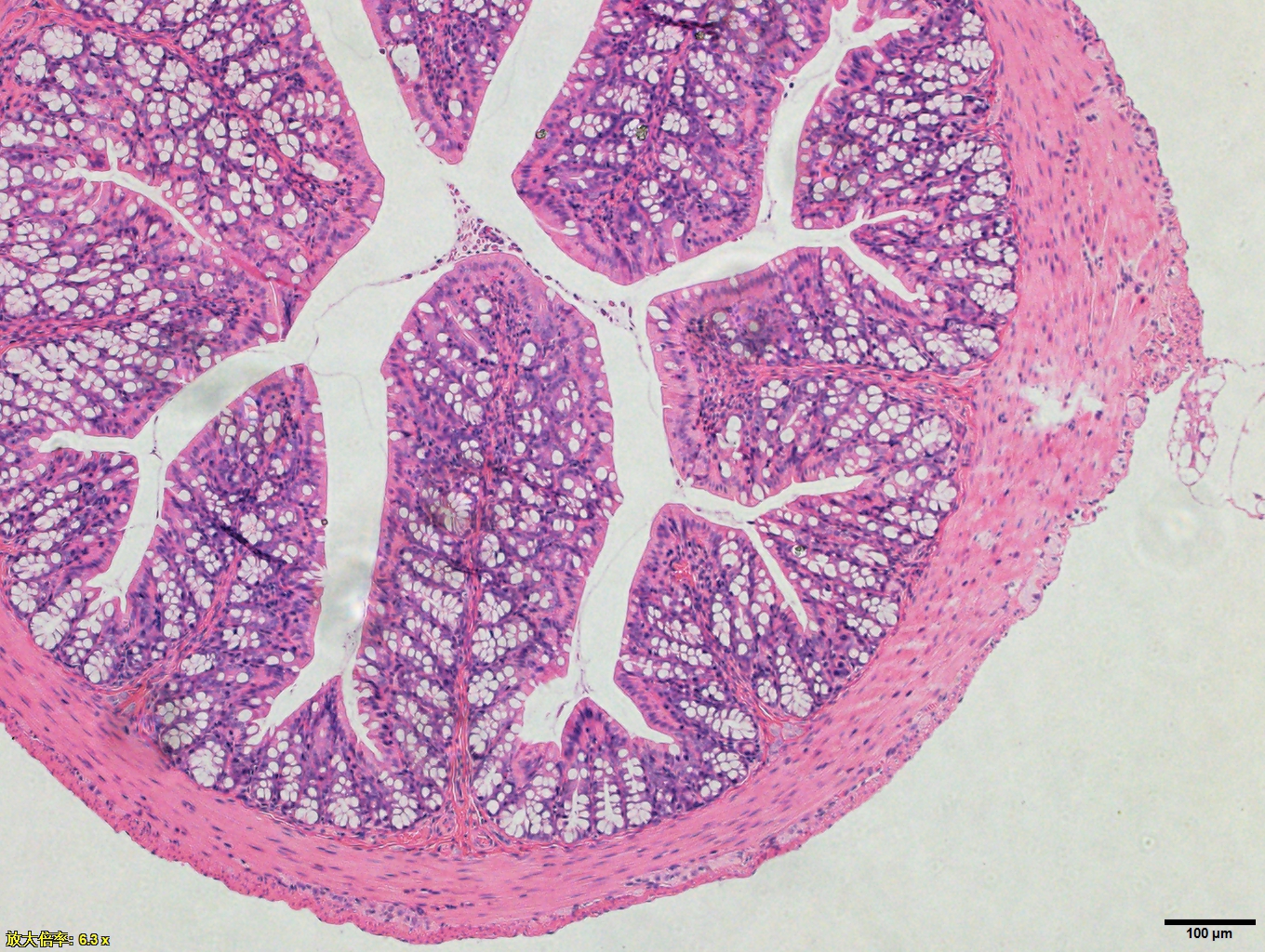

Supplement: Supplementary file 1 [file Data_Sheet_1.zip › Figure 3/DSS+MOLP-L 100x.jpg]

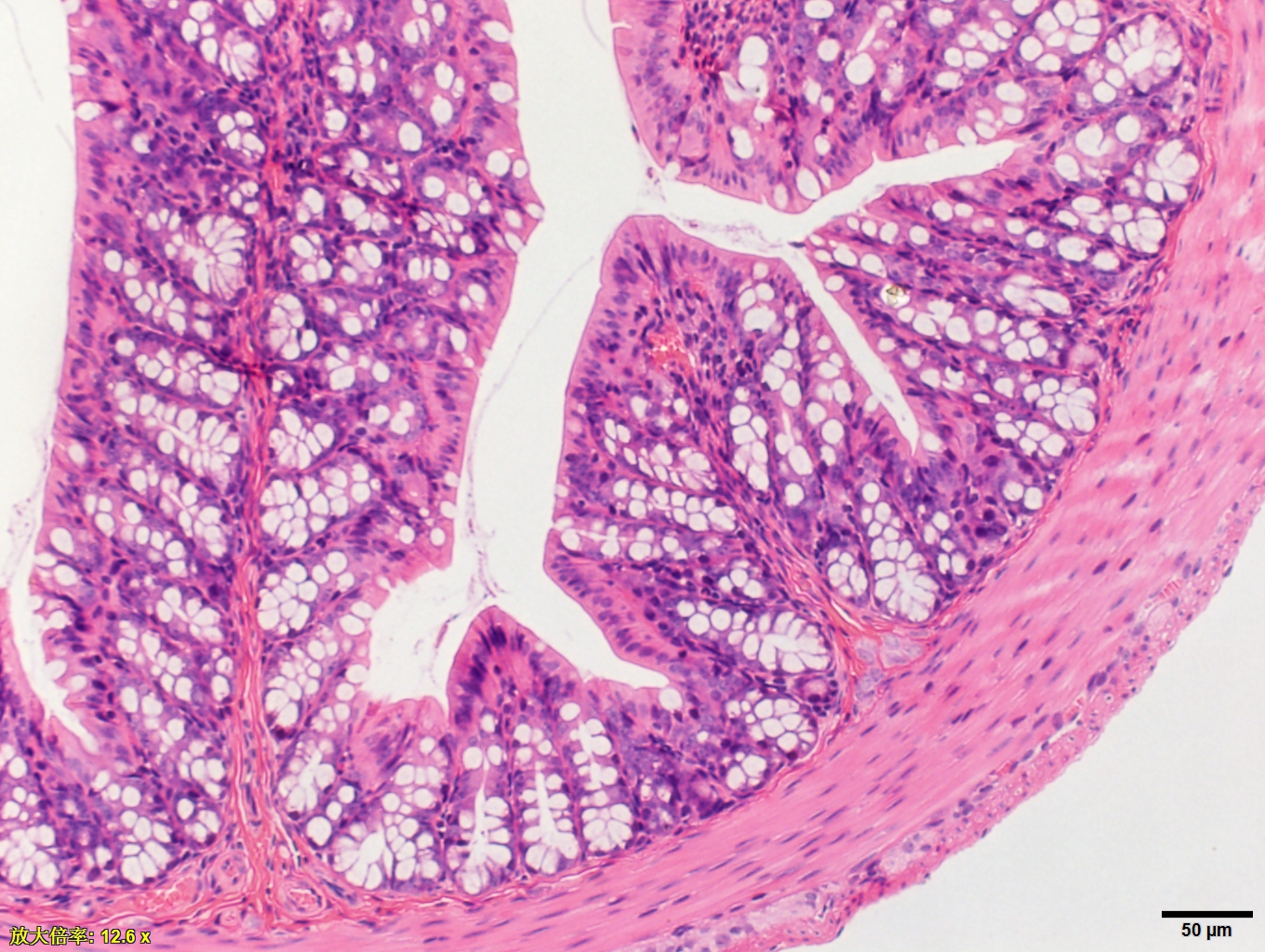

Supplement: Supplementary file 1 [file Data_Sheet_1.zip › Figure 3/DSS+MOLP-L.jpg]

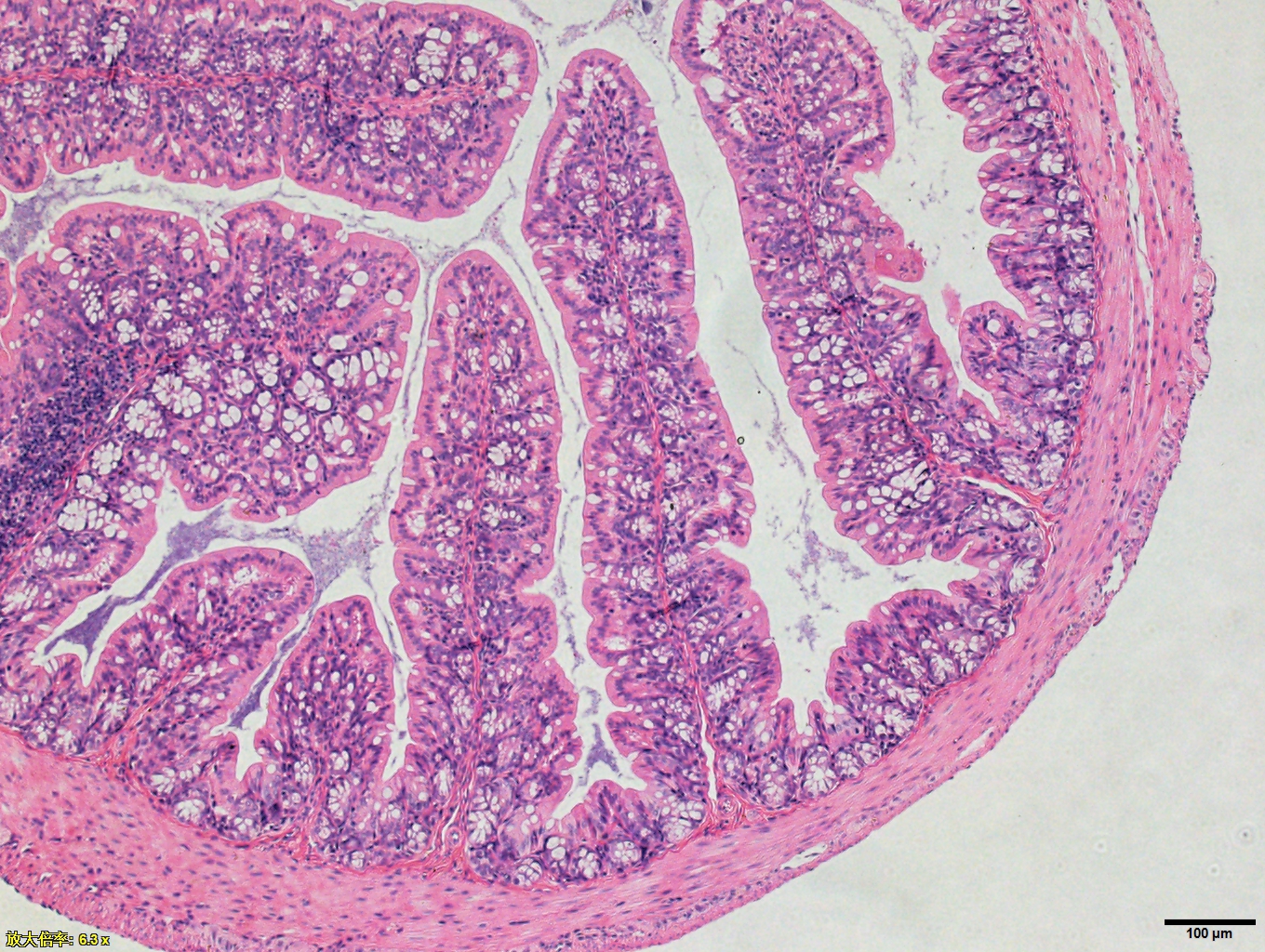

Supplement: Supplementary file 1 [file Data_Sheet_1.zip › Figure 3/DSS+MOLP-M 100×.jpg]

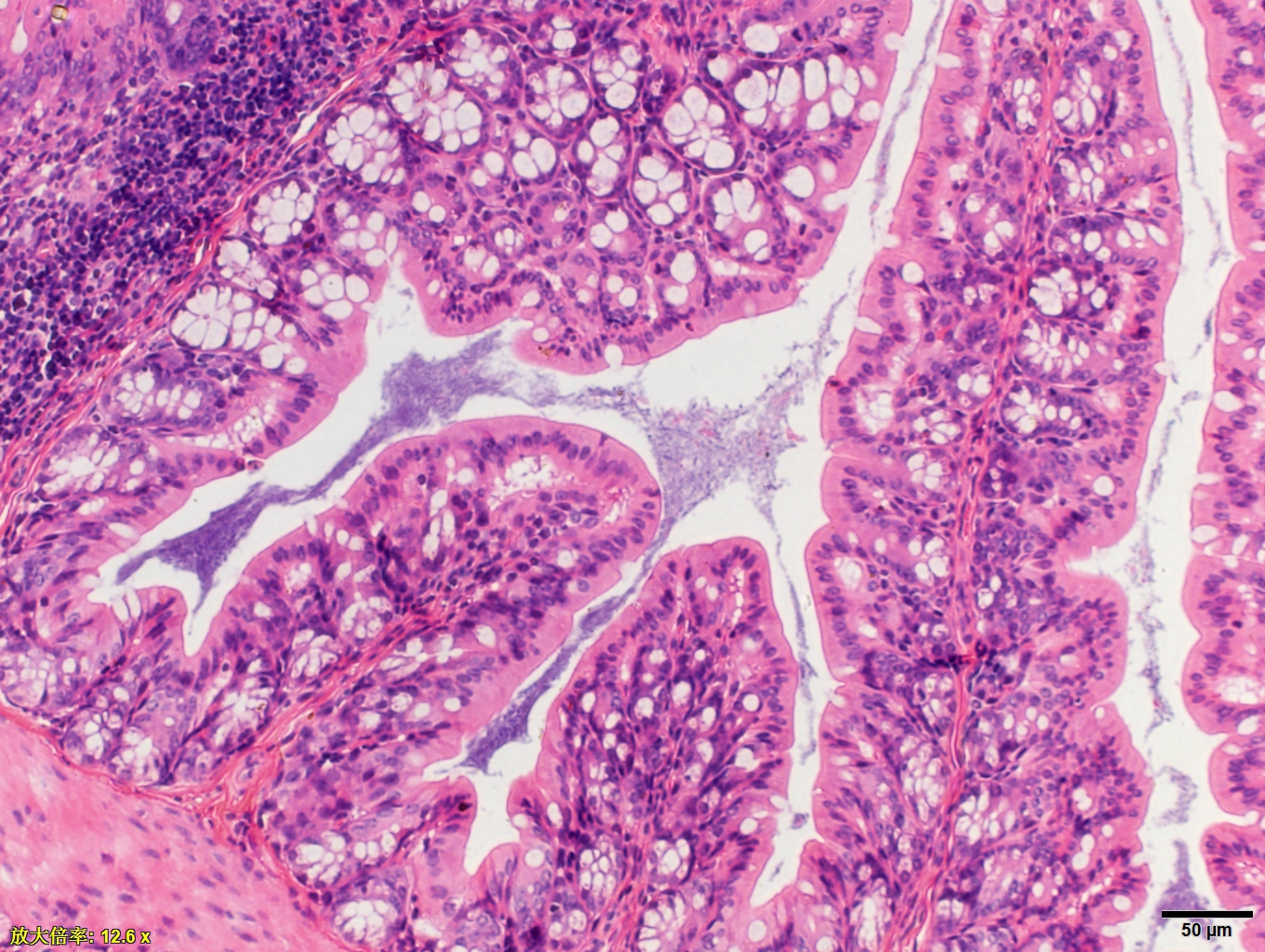

Supplement: Supplementary file 1 [file Data_Sheet_1.zip › Figure 3/DSS+MOLP-M 200x.jpg]

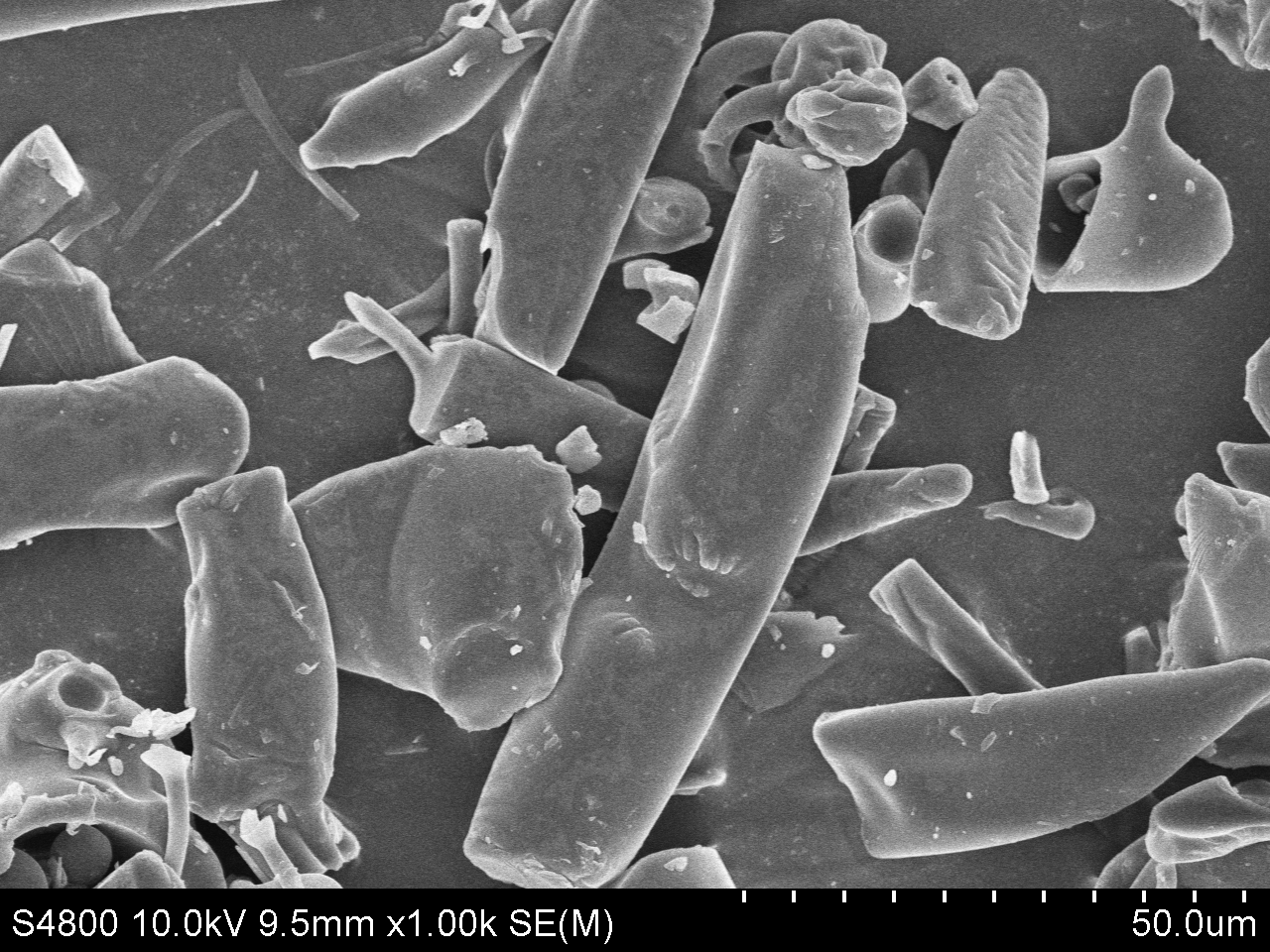

Supplement: Supplementary file 1 [file Data_Sheet_1.zip › Figure 2/Fig 2F.tif]

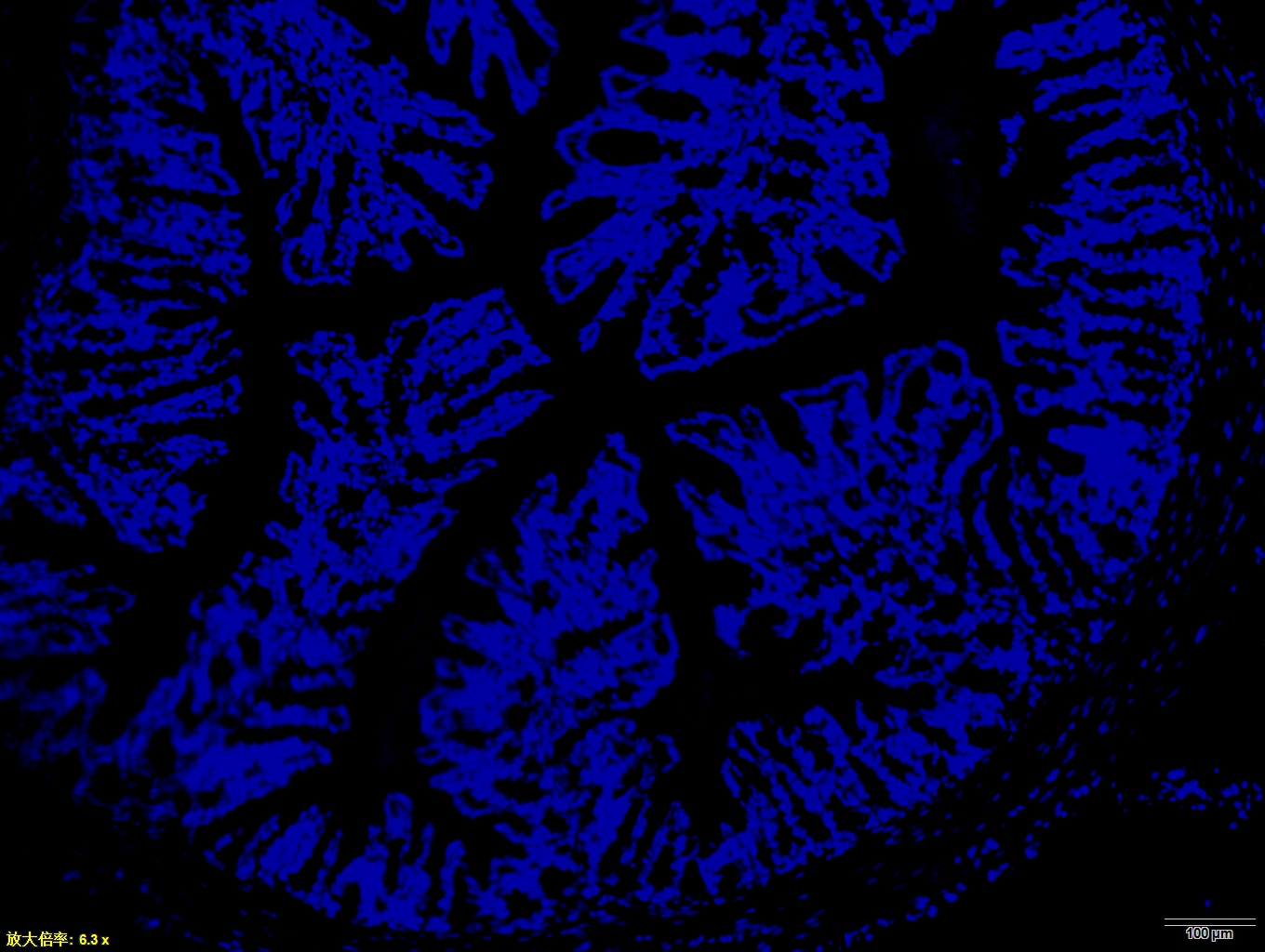

Supplement: Supplementary file 2 [file Data_Sheet_2.zip › Figure 6/Con Occludin DAPI .jpg]

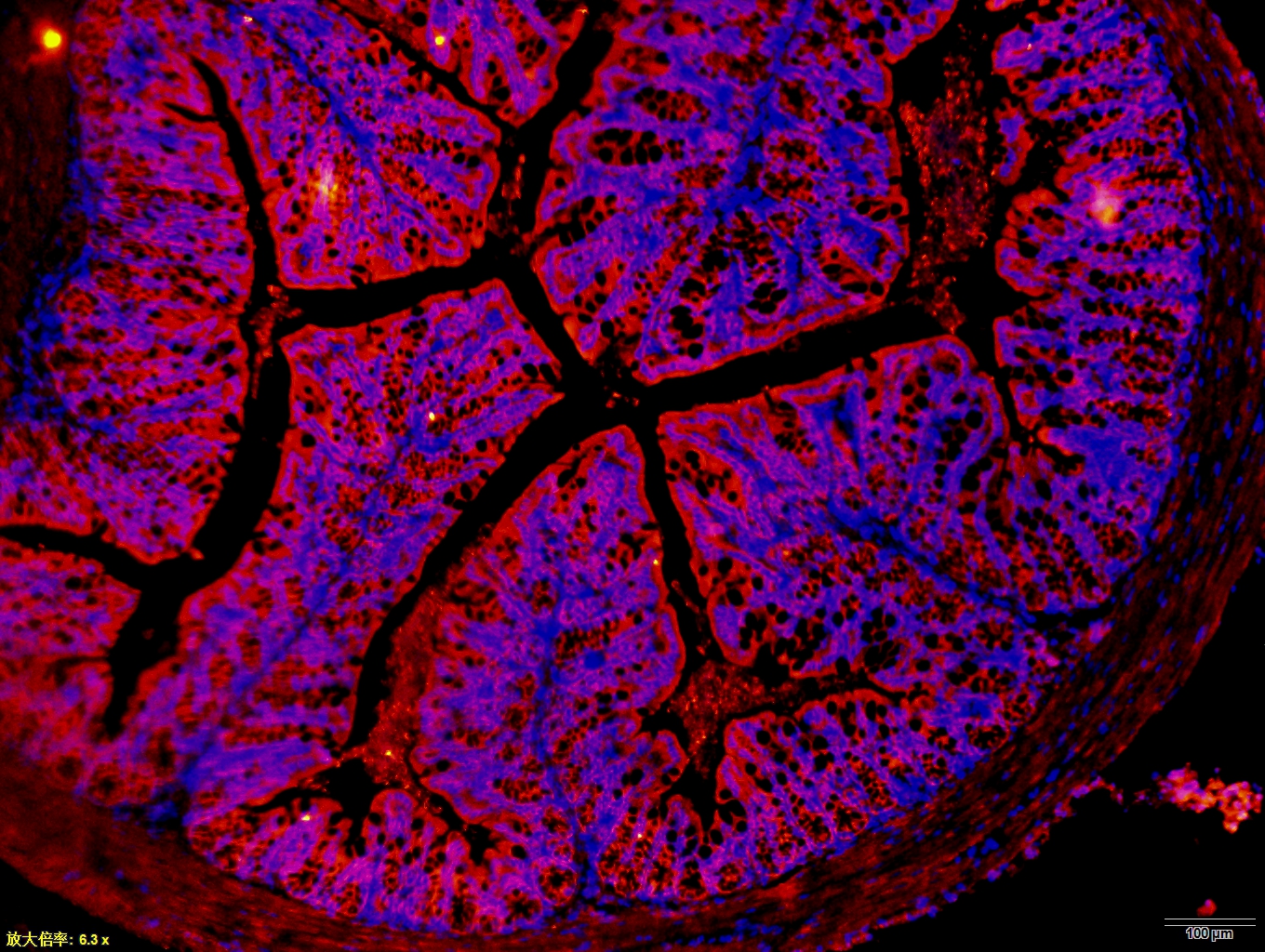

Supplement: Supplementary file 2 [file Data_Sheet_2.zip › Figure 6/Con Occludin MERGE 2.jpg]

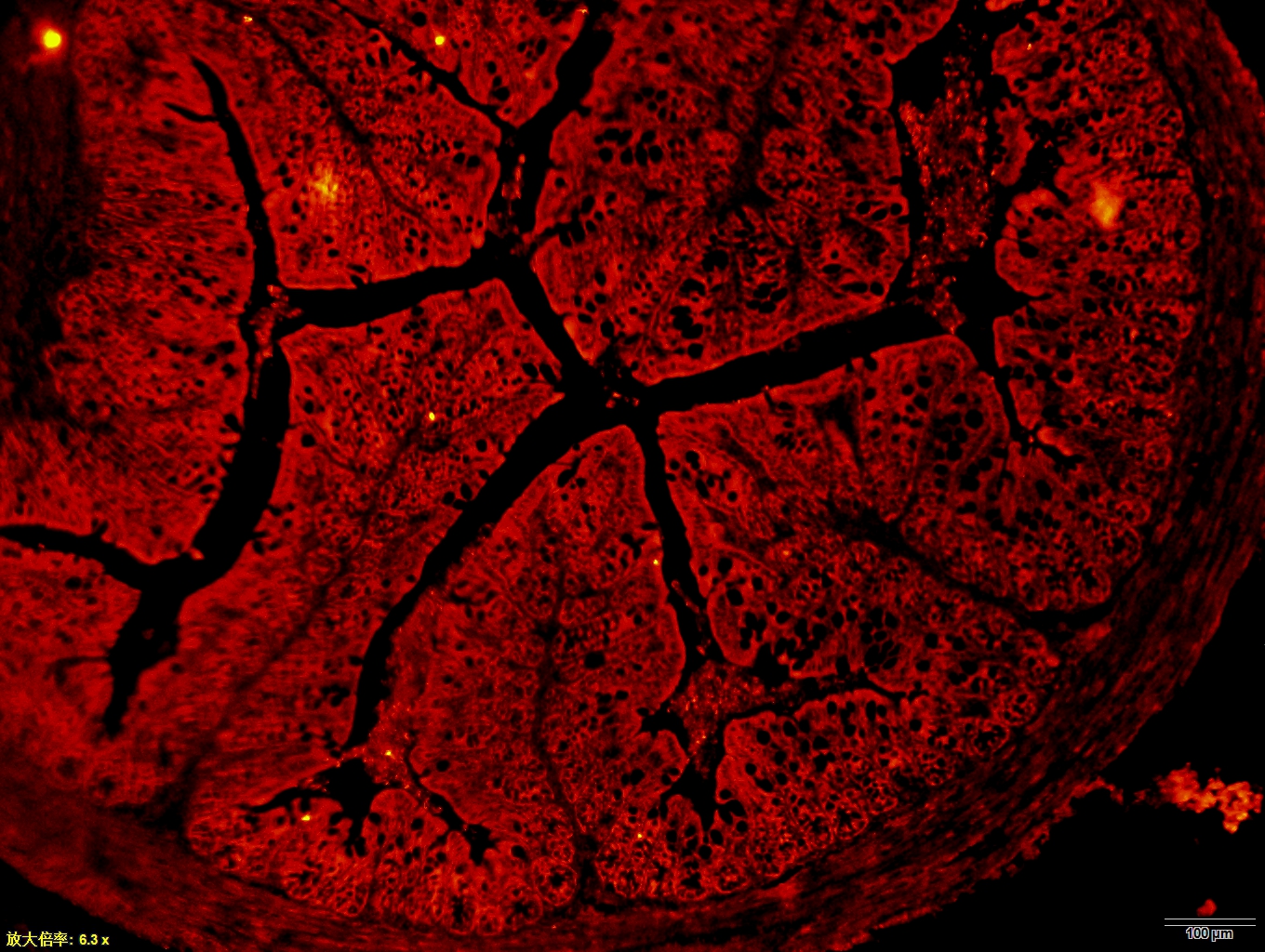

Supplement: Supplementary file 2 [file Data_Sheet_2.zip › Figure 6/Con Occludin.jpg]

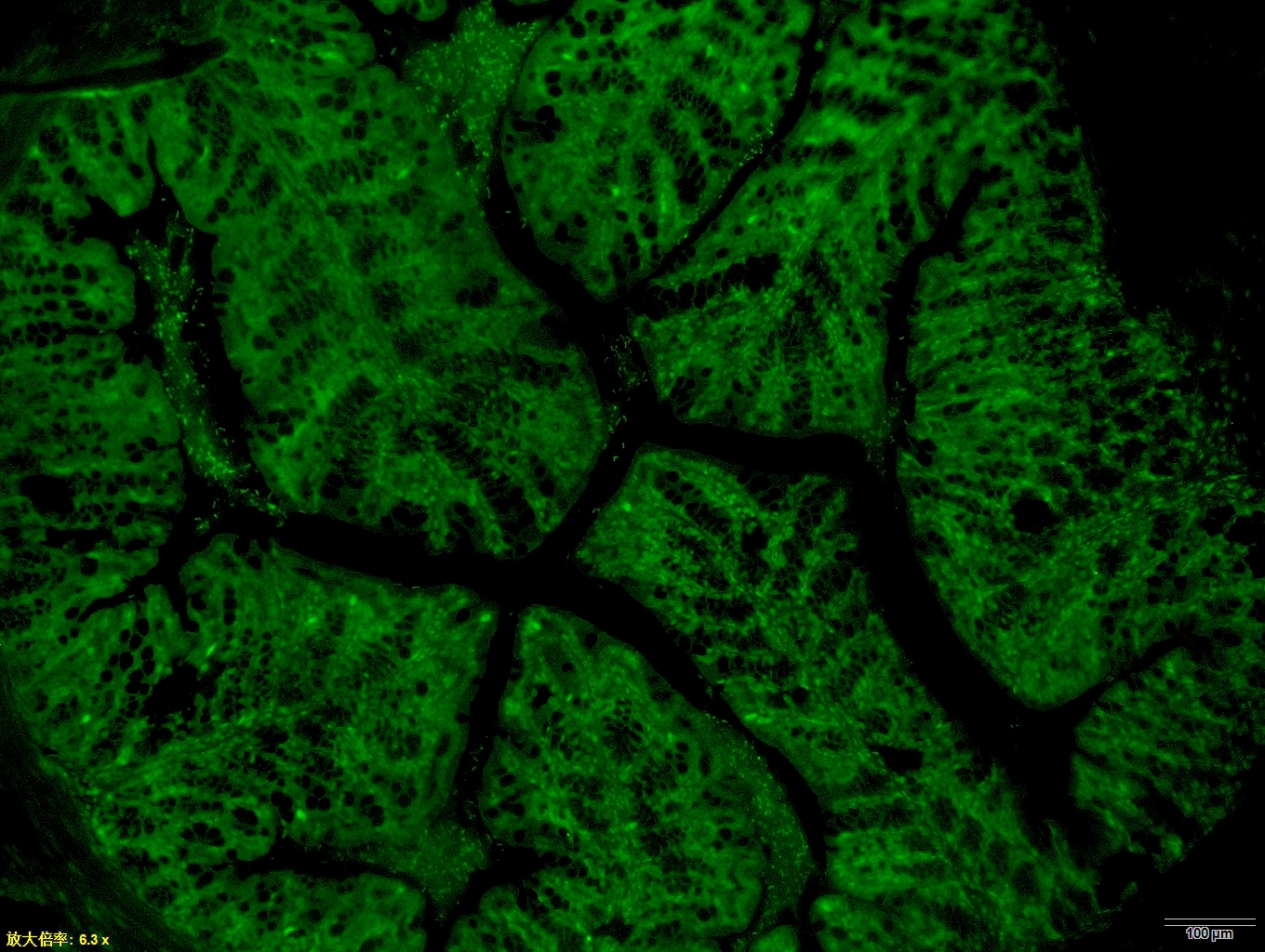

Supplement: Supplementary file 2 [file Data_Sheet_2.zip › Figure 6/Con ZO-1 .jpg]

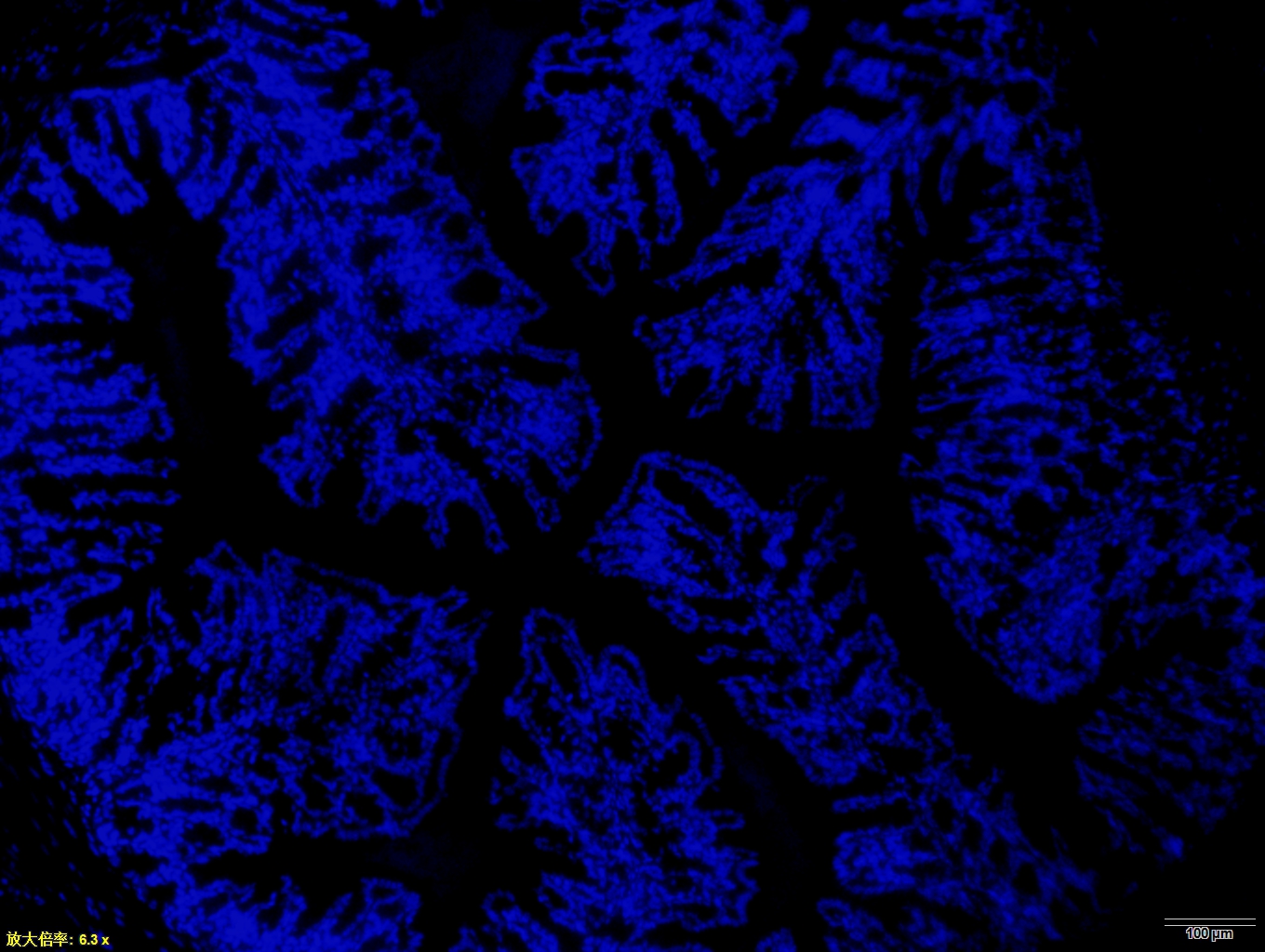

Supplement: Supplementary file 2 [file Data_Sheet_2.zip › Figure 6/Con ZO-1 DAPI.jpg]

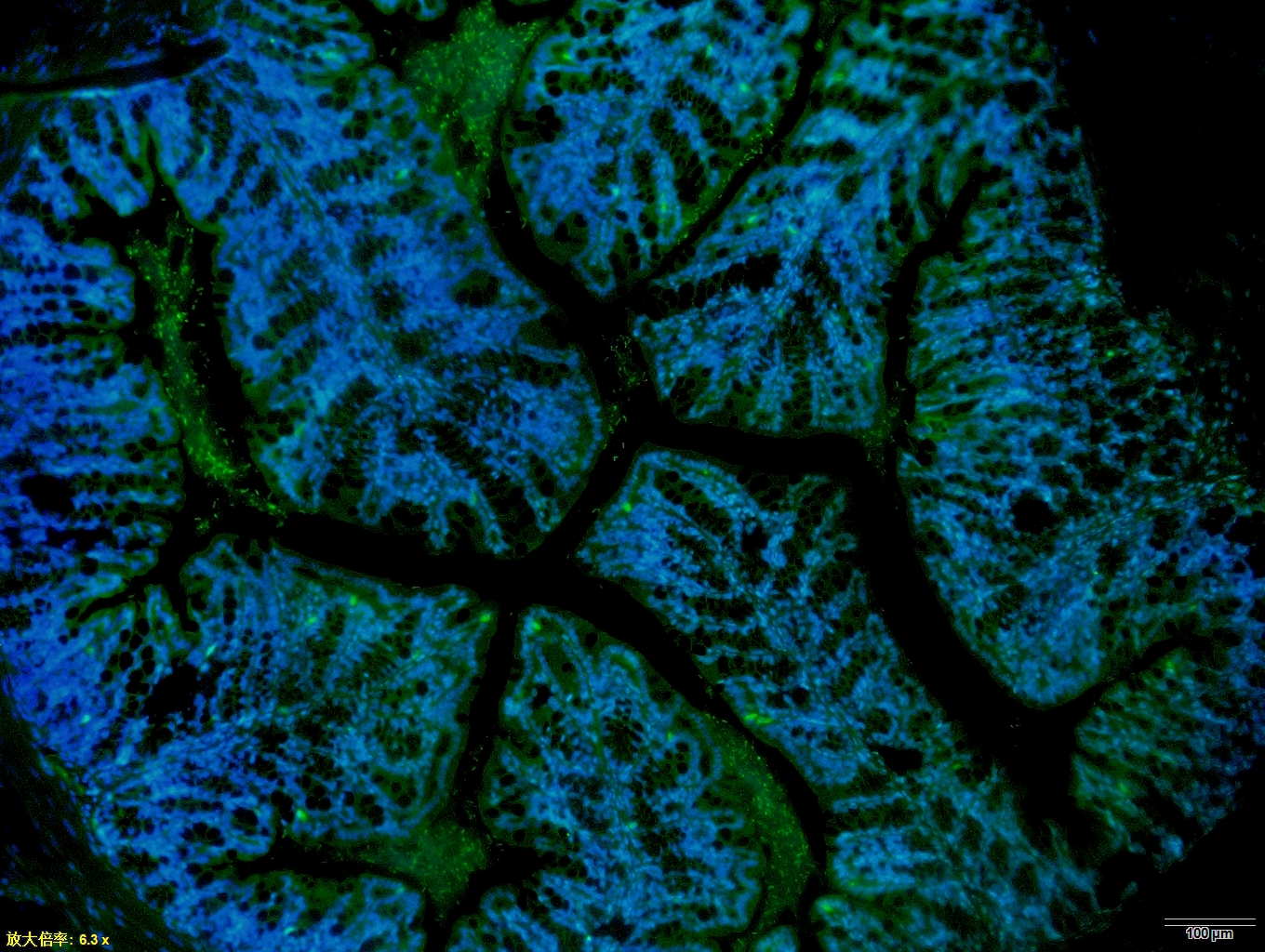

Supplement: Supplementary file 2 [file Data_Sheet_2.zip › Figure 6/Con ZO-1 merge .jpg]

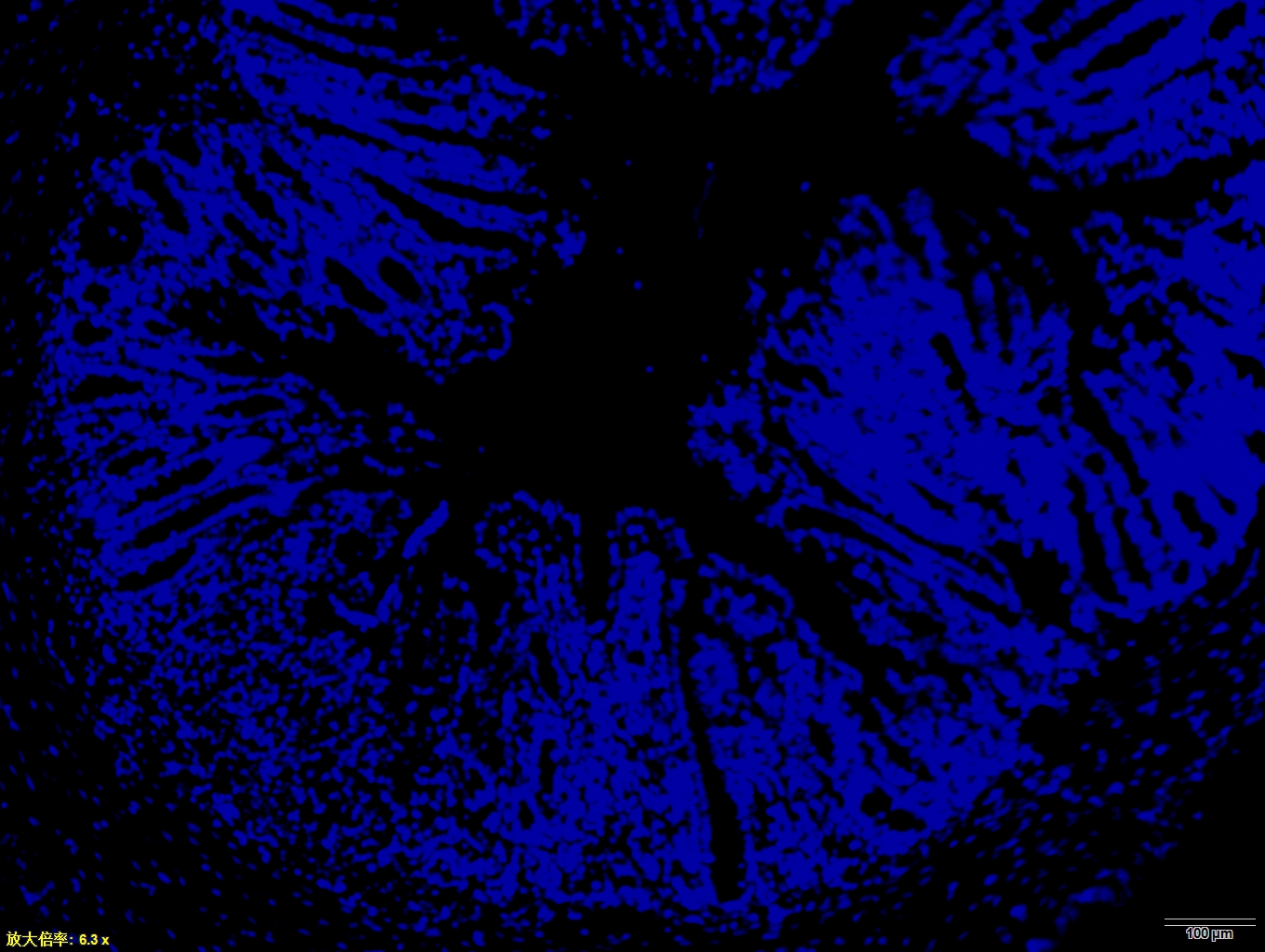

Supplement: Supplementary file 2 [file Data_Sheet_2.zip › Figure 6/DSS Occludin DAPI .jpg]

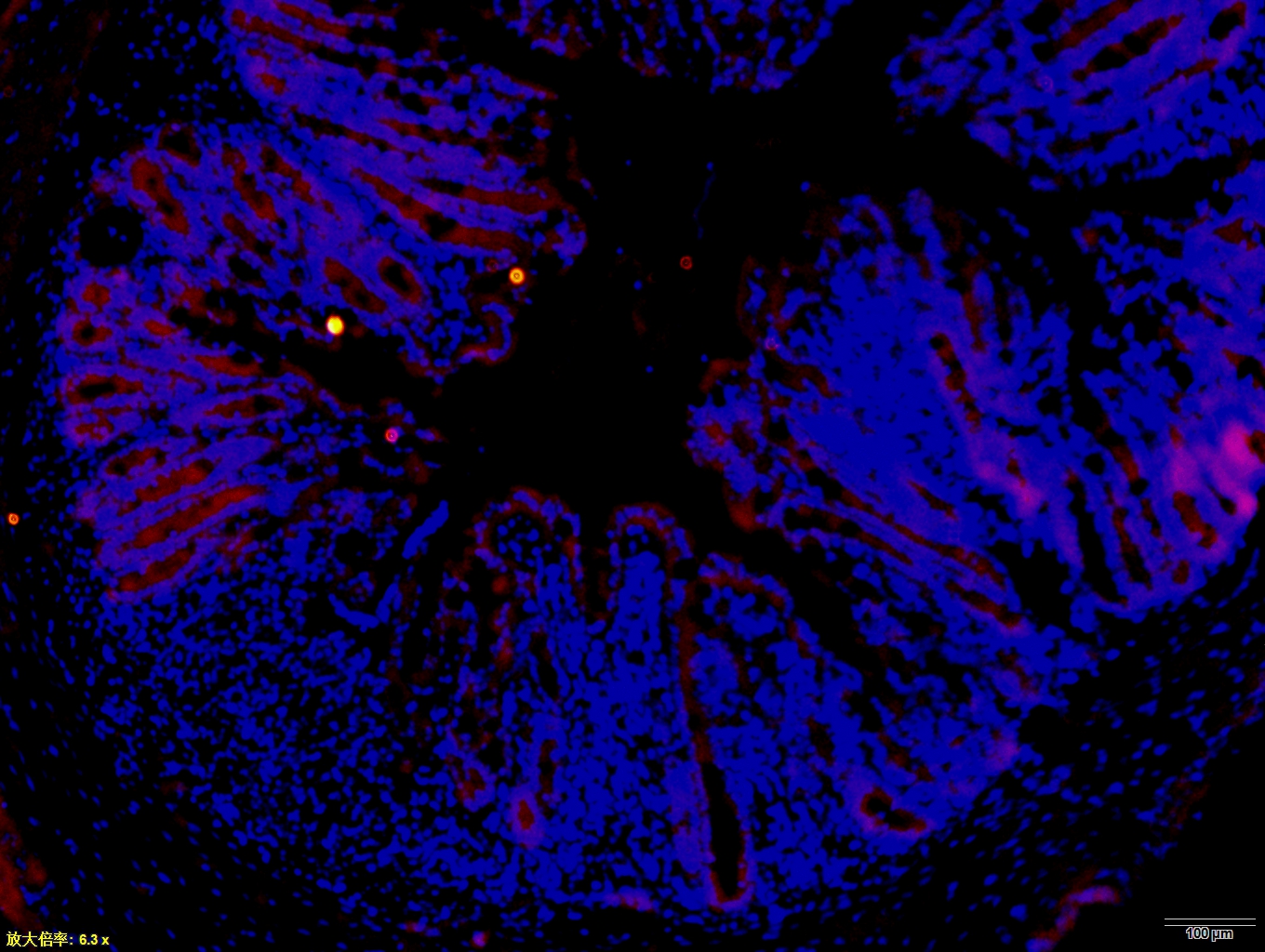

Supplement: Supplementary file 2 [file Data_Sheet_2.zip › Figure 6/DSS Occludin MERGE .jpg]

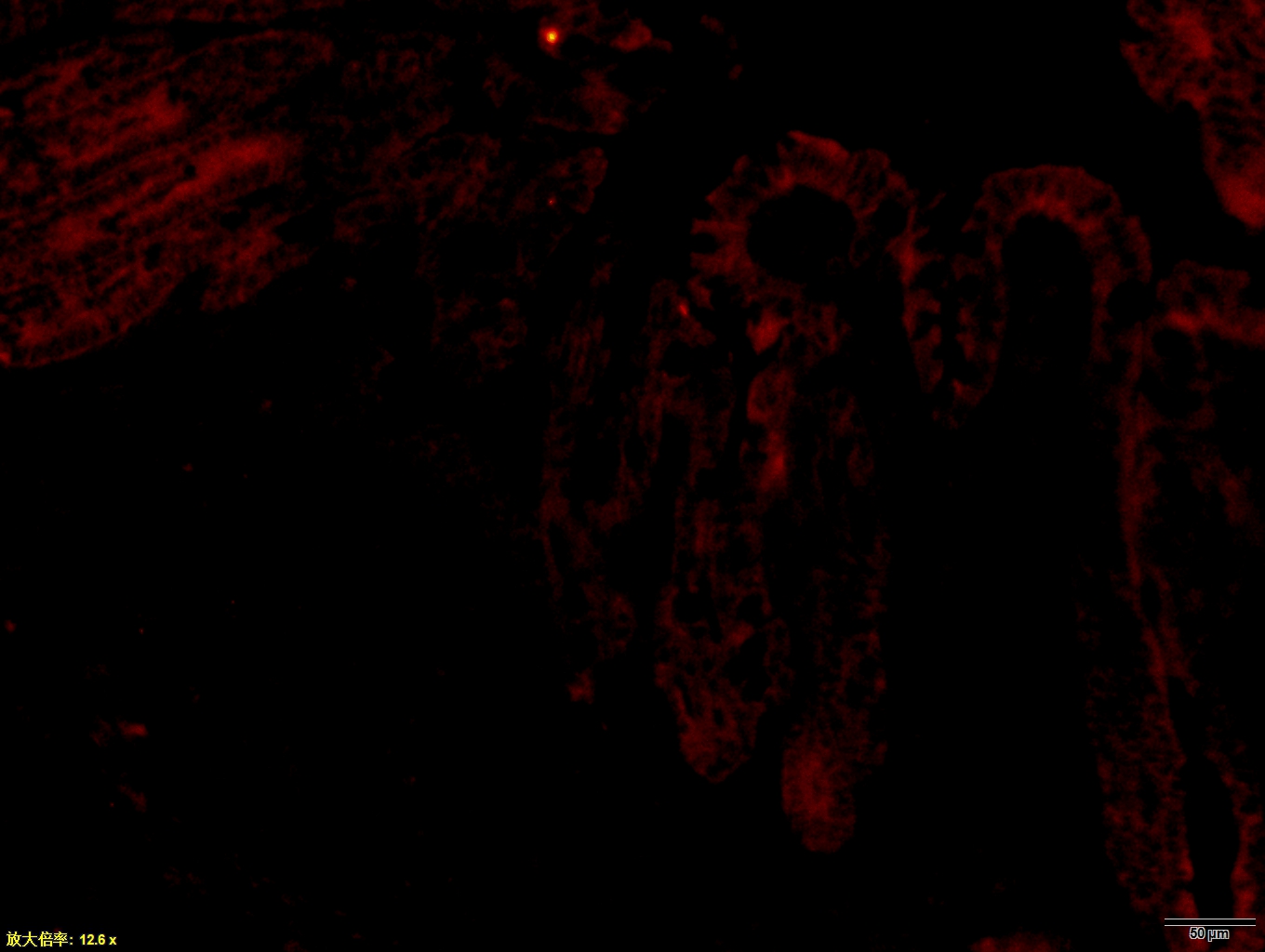

Supplement: Supplementary file 2 [file Data_Sheet_2.zip › Figure 6/DSS Occludin.jpg]

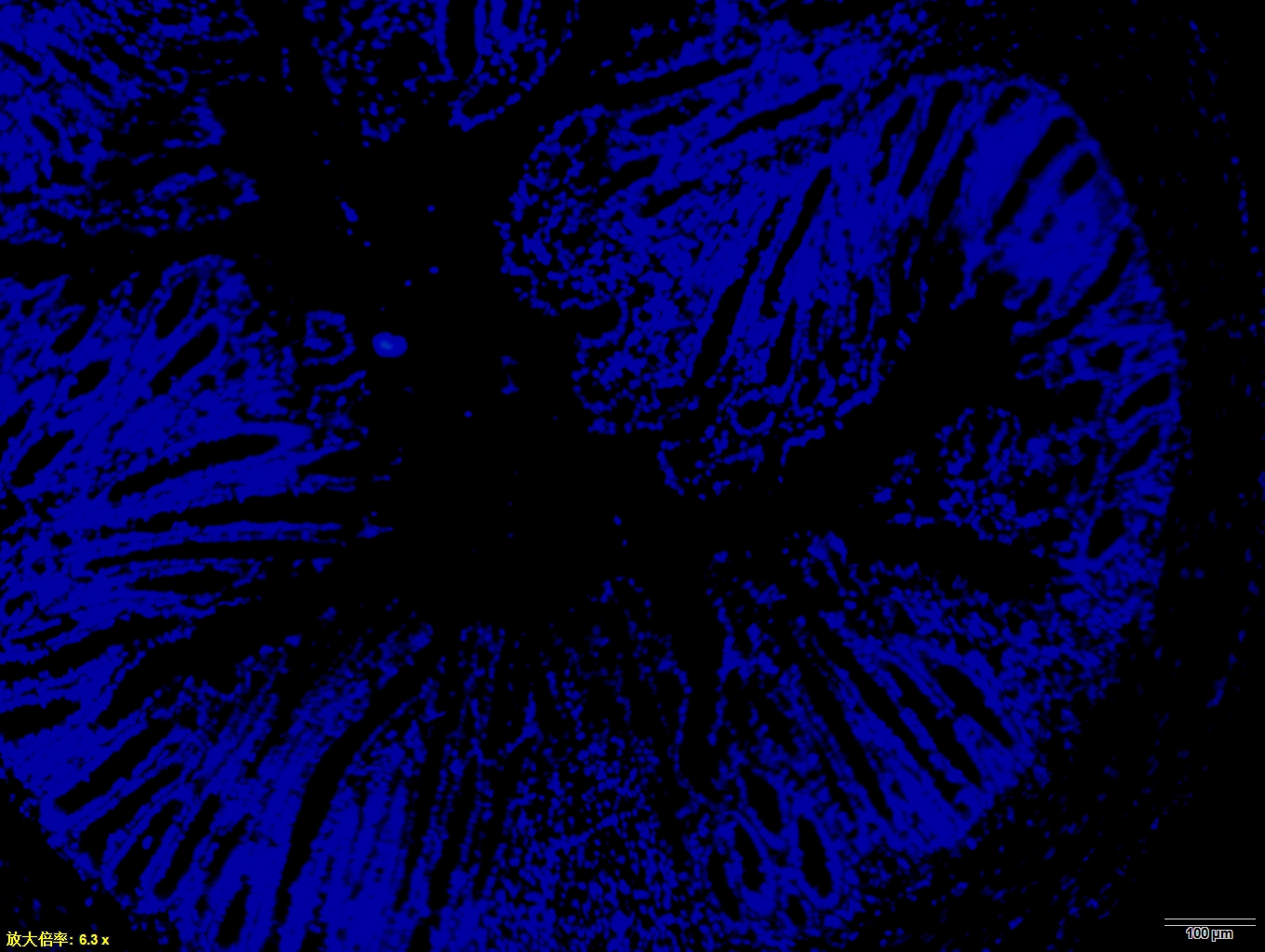

Supplement: Supplementary file 2 [file Data_Sheet_2.zip › Figure 6/DSS ZO-1 DAPI .jpg]

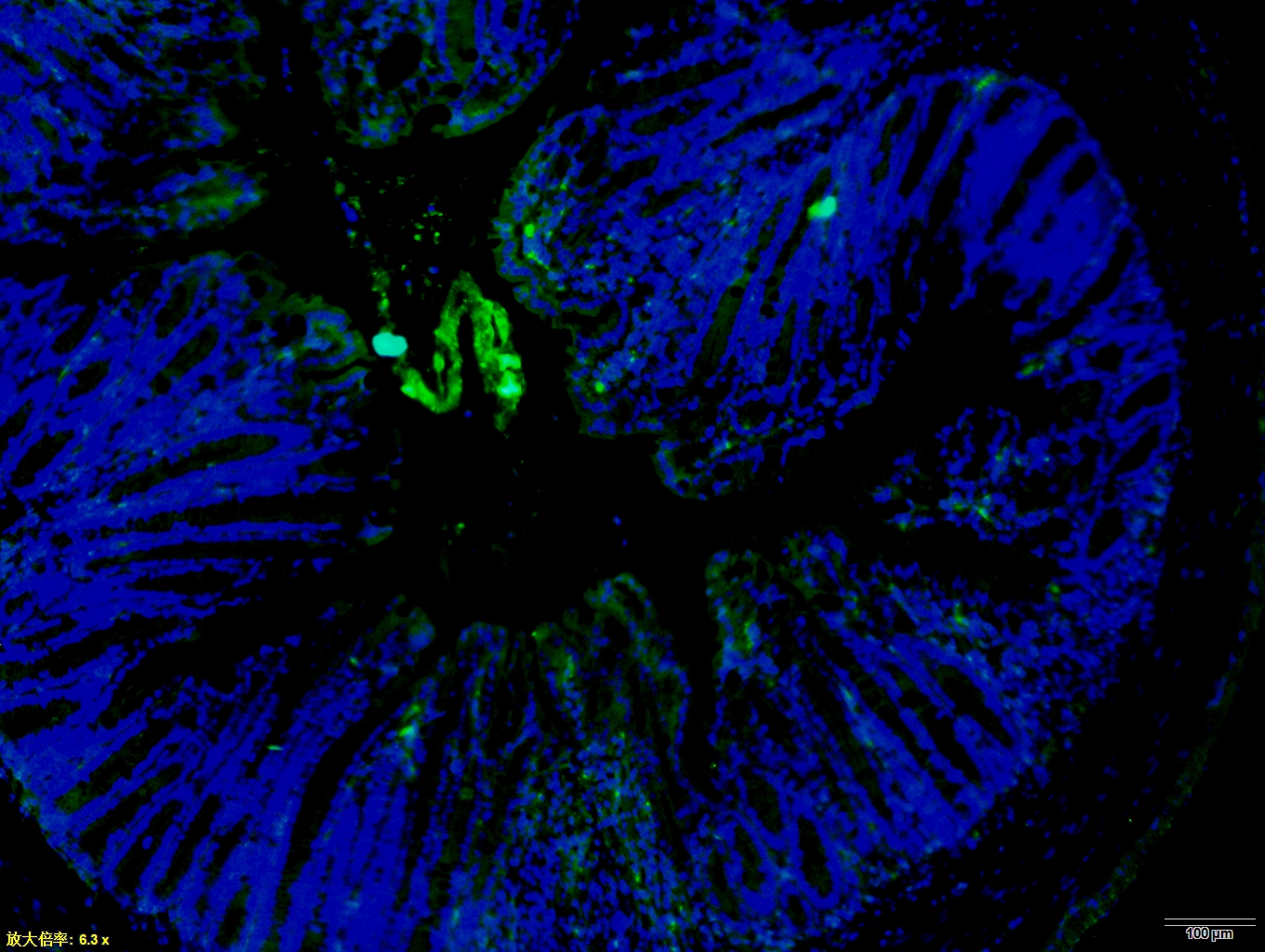

Supplement: Supplementary file 2 [file Data_Sheet_2.zip › Figure 6/DSS ZO-1 merge .jpg]

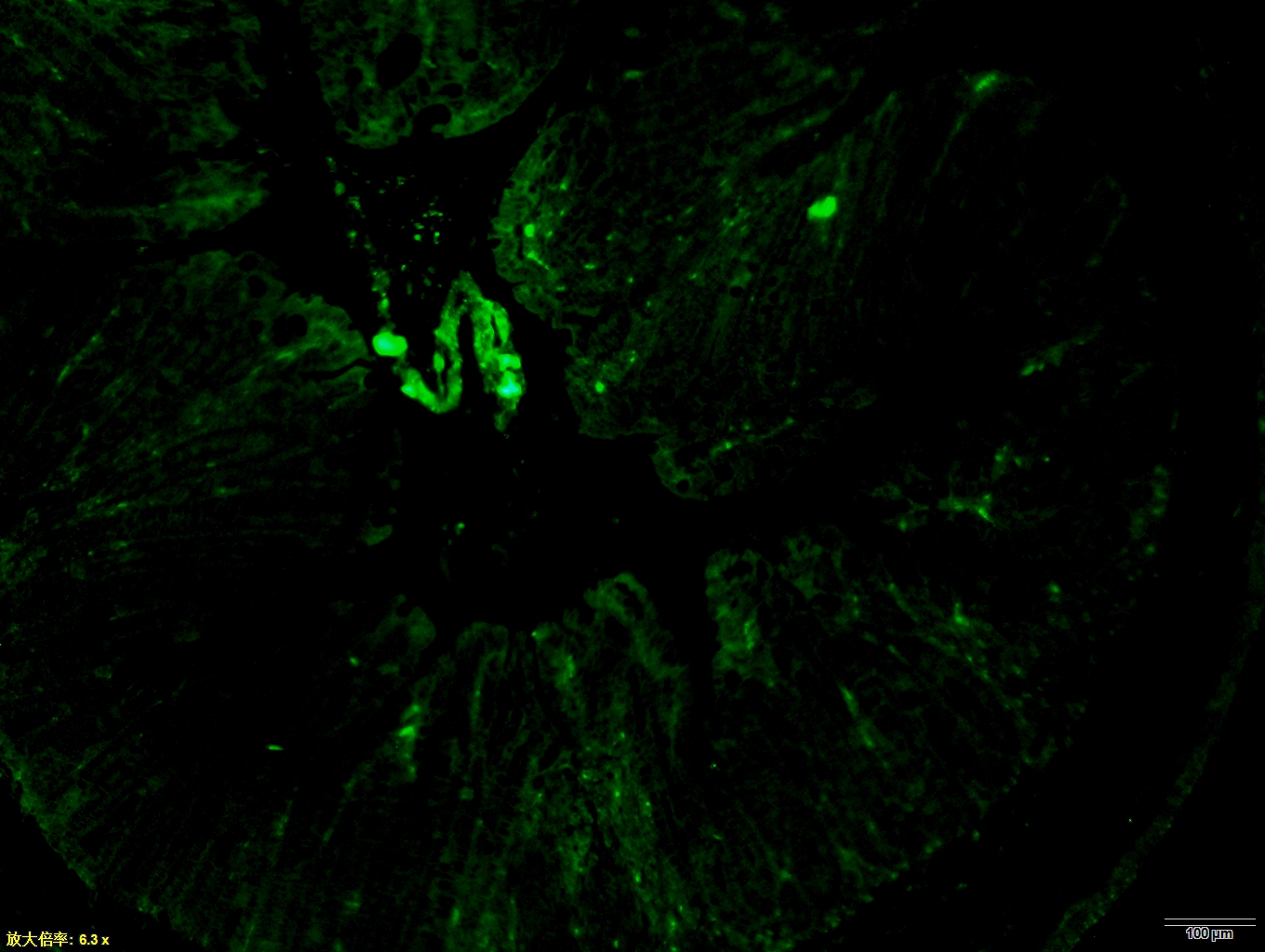

Supplement: Supplementary file 2 [file Data_Sheet_2.zip › Figure 6/DSS ZO-1.jpg]

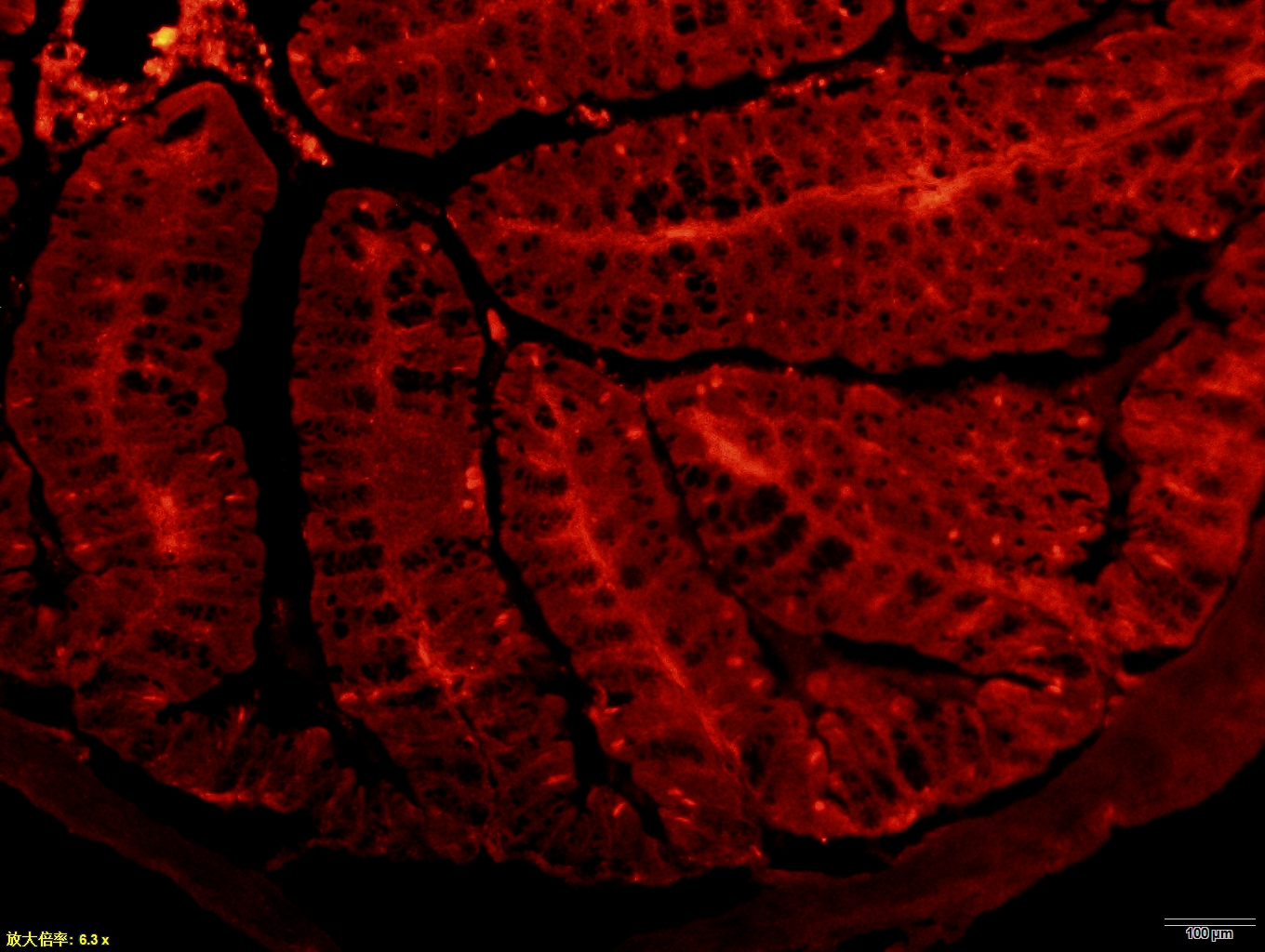

Supplement: Supplementary file 2 [file Data_Sheet_2.zip › Figure 6/DSS+MOLP-H Occludin .jpg]

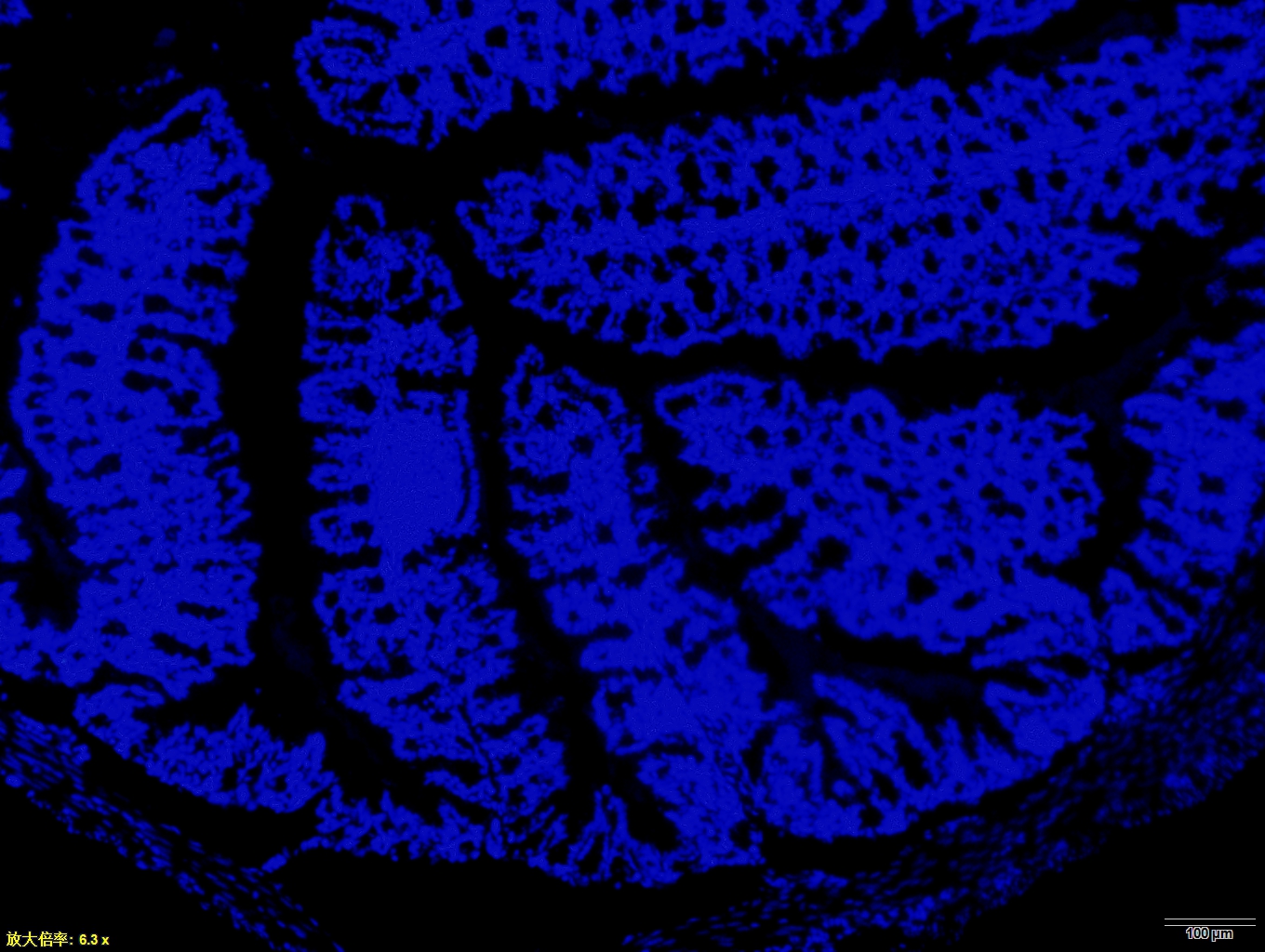

Supplement: Supplementary file 2 [file Data_Sheet_2.zip › Figure 6/DSS+MOLP-H Occludin DAPI .jpg]

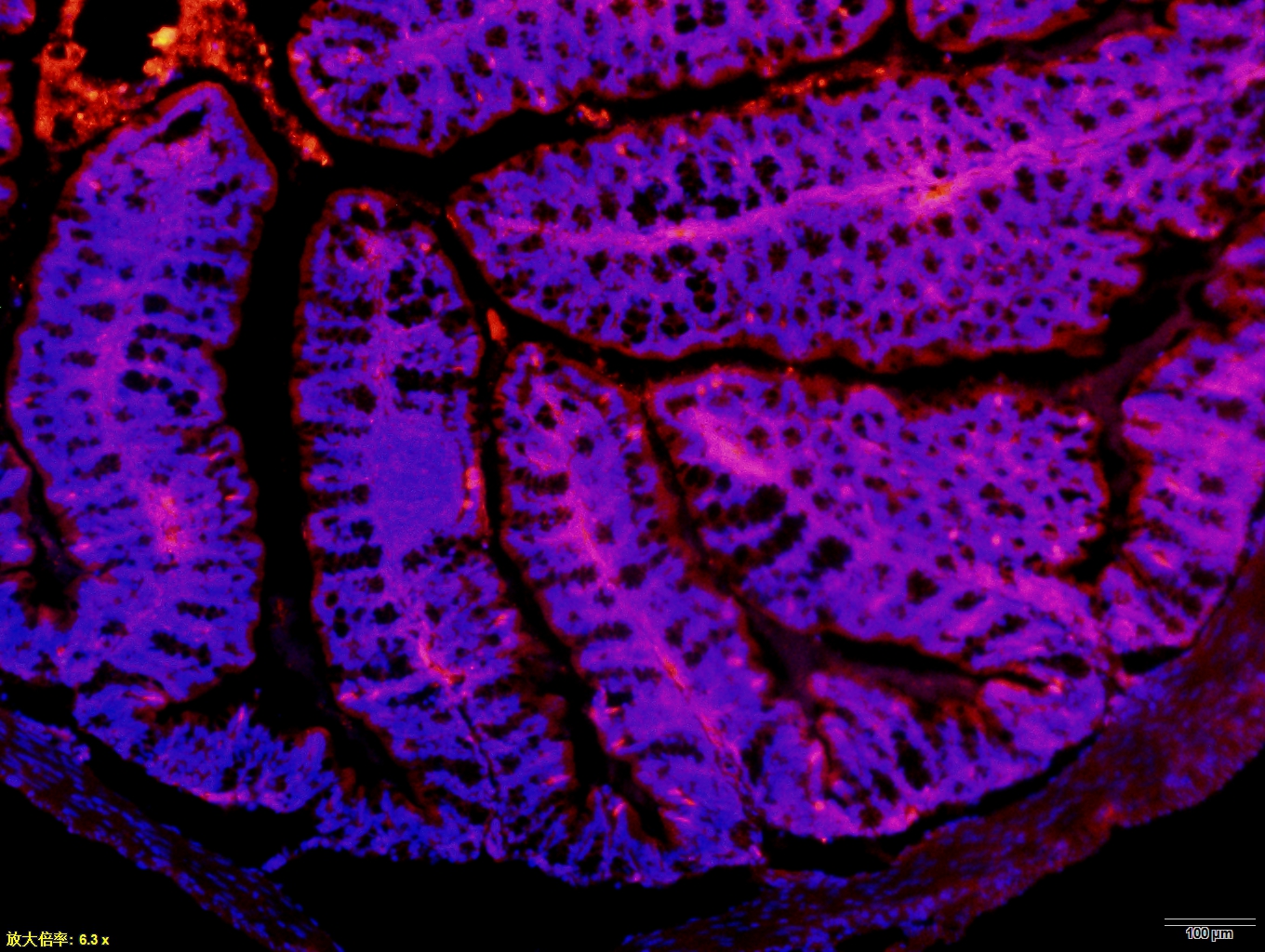

Supplement: Supplementary file 2 [file Data_Sheet_2.zip › Figure 6/DSS+MOLP-H Occludin merge .jpg]

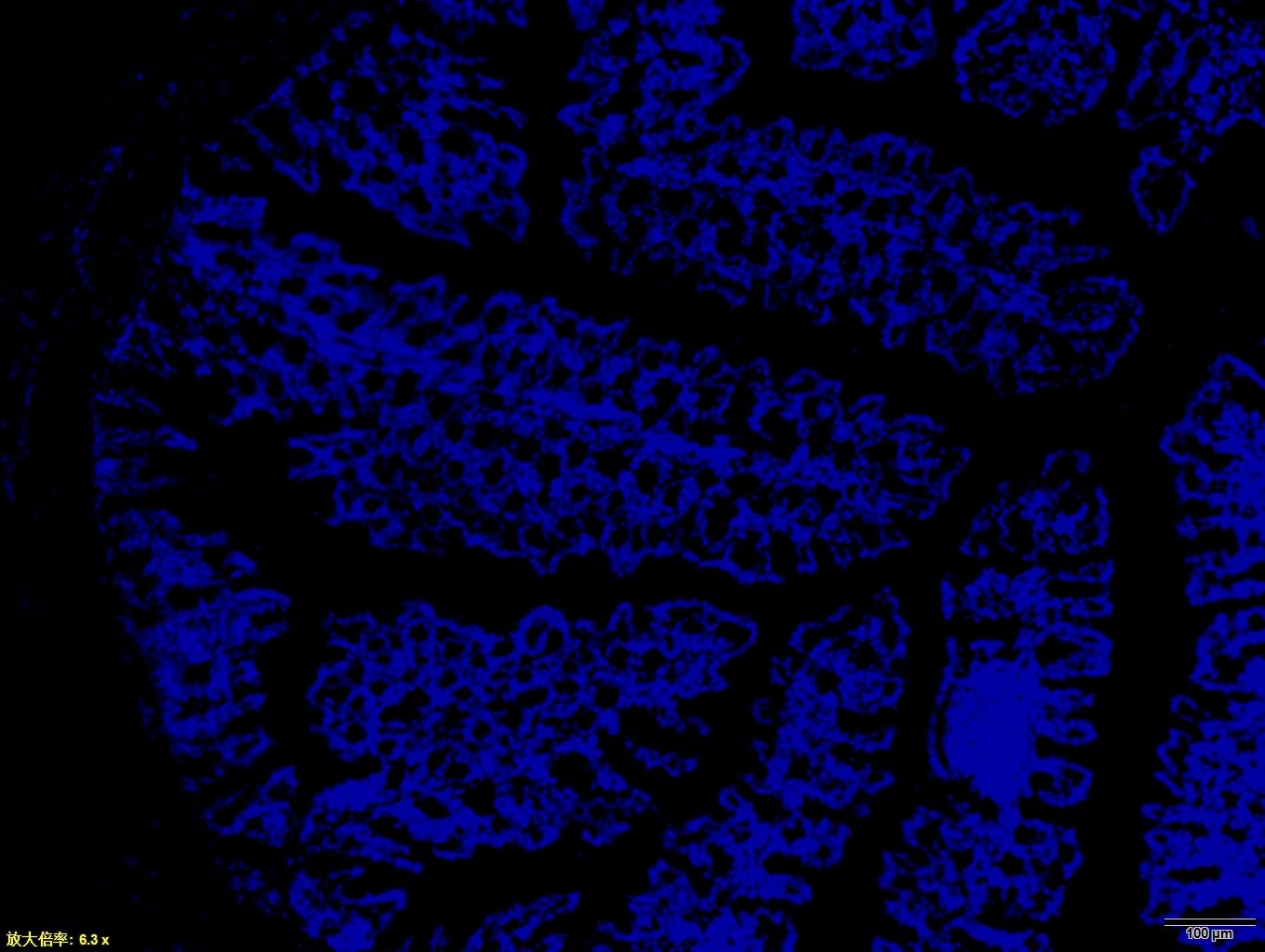

Supplement: Supplementary file 2 [file Data_Sheet_2.zip › Figure 6/DSS+MOLP-H ZO-1 DIPA.jpg]

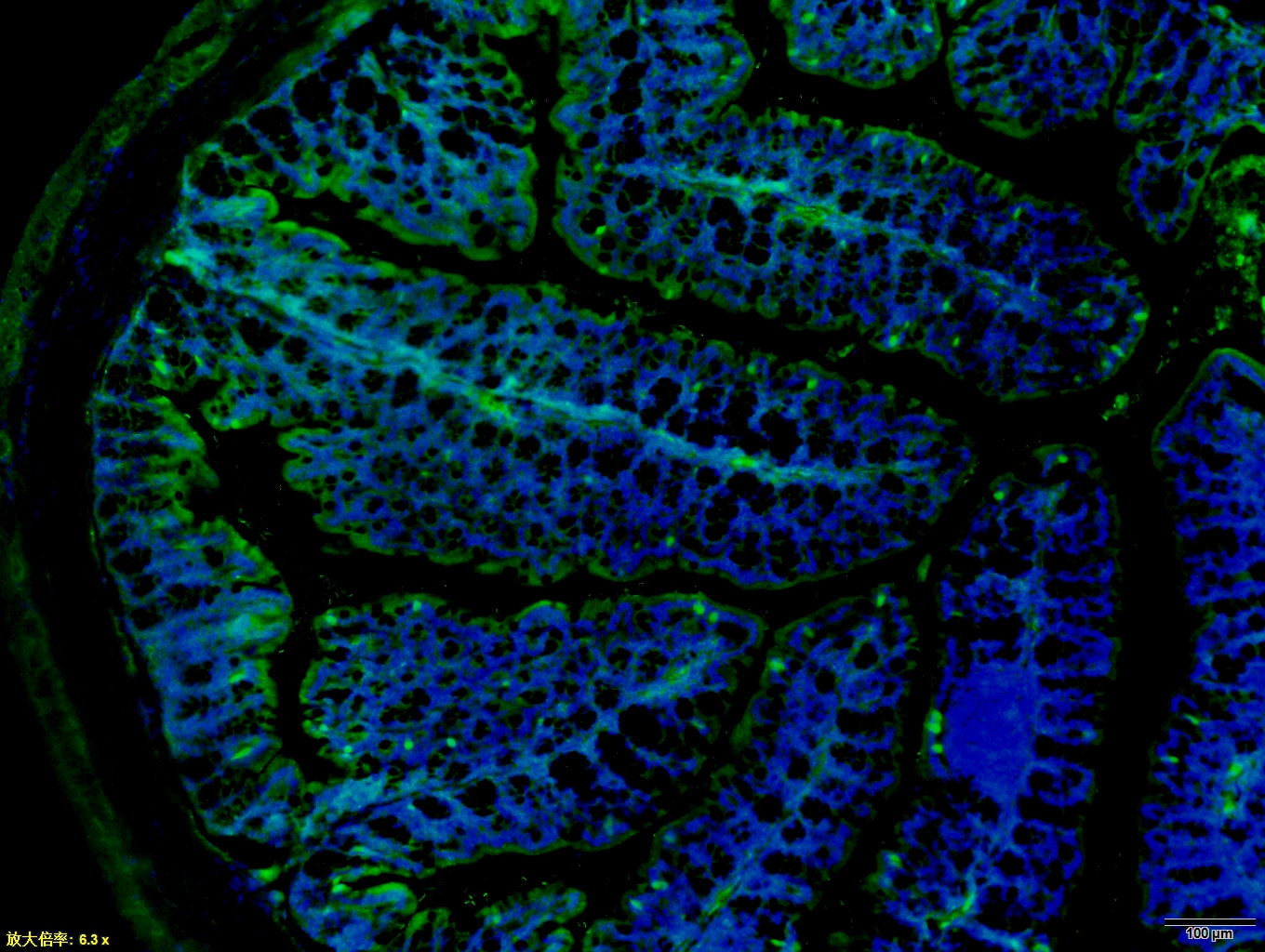

Supplement: Supplementary file 2 [file Data_Sheet_2.zip › Figure 6/DSS+MOLP-H ZO-1 MERGE.jpg]

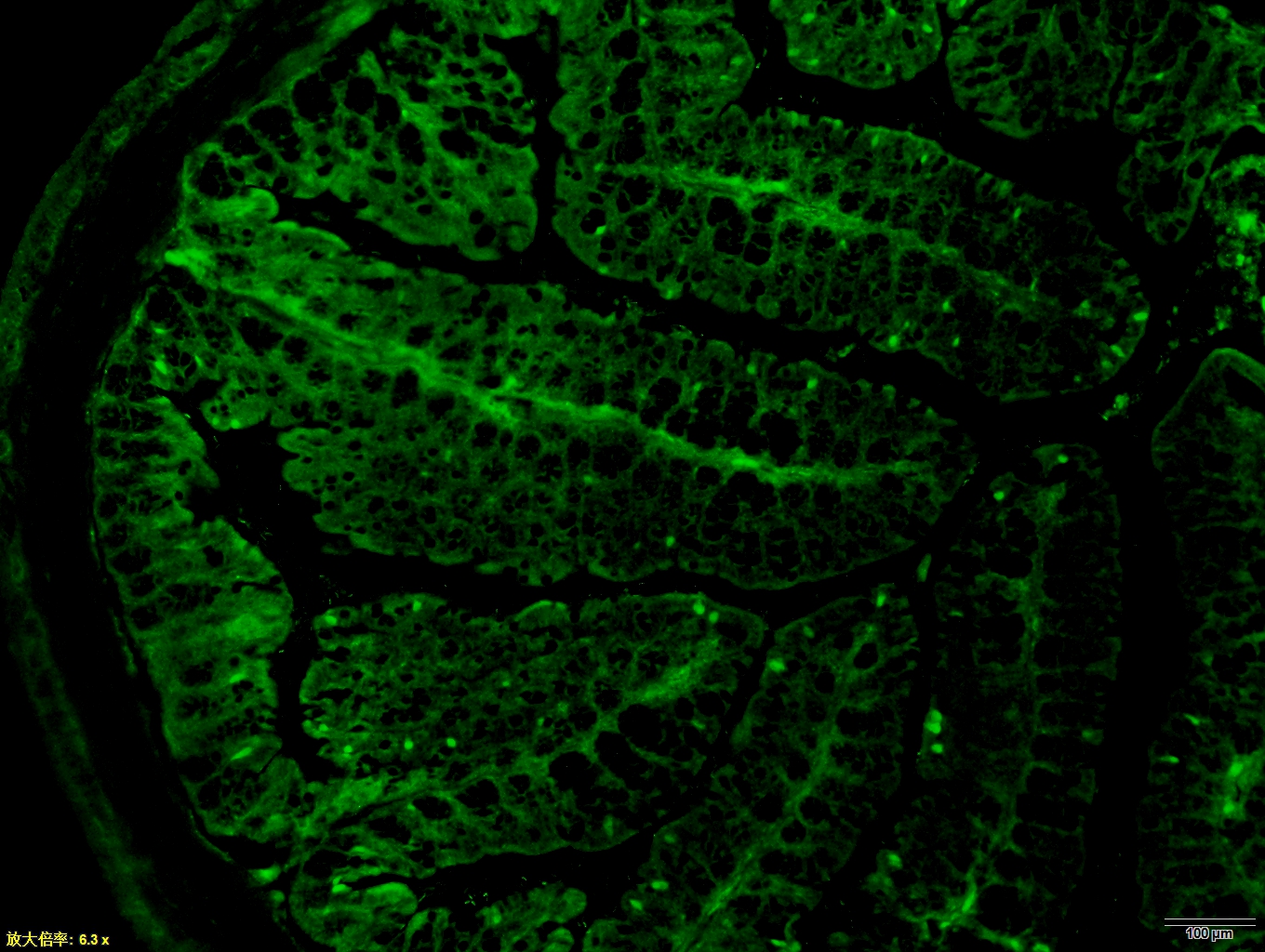

Supplement: Supplementary file 2 [file Data_Sheet_2.zip › Figure 6/DSS+MOLP-H zo-1.jpg]

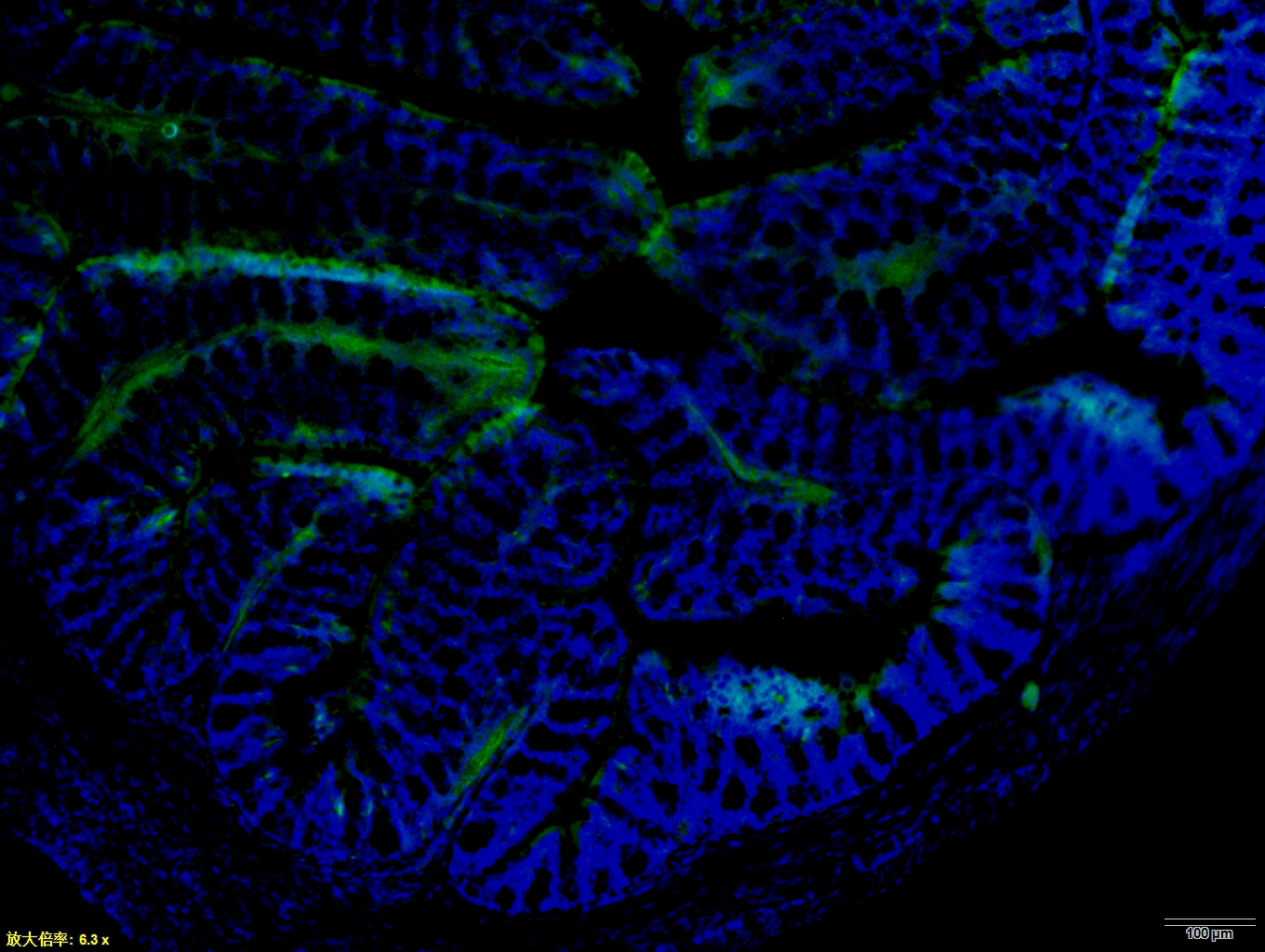

Supplement: Supplementary file 2 [file Data_Sheet_2.zip › Figure 6/DSS+MOLP-L MERGE.jpg]

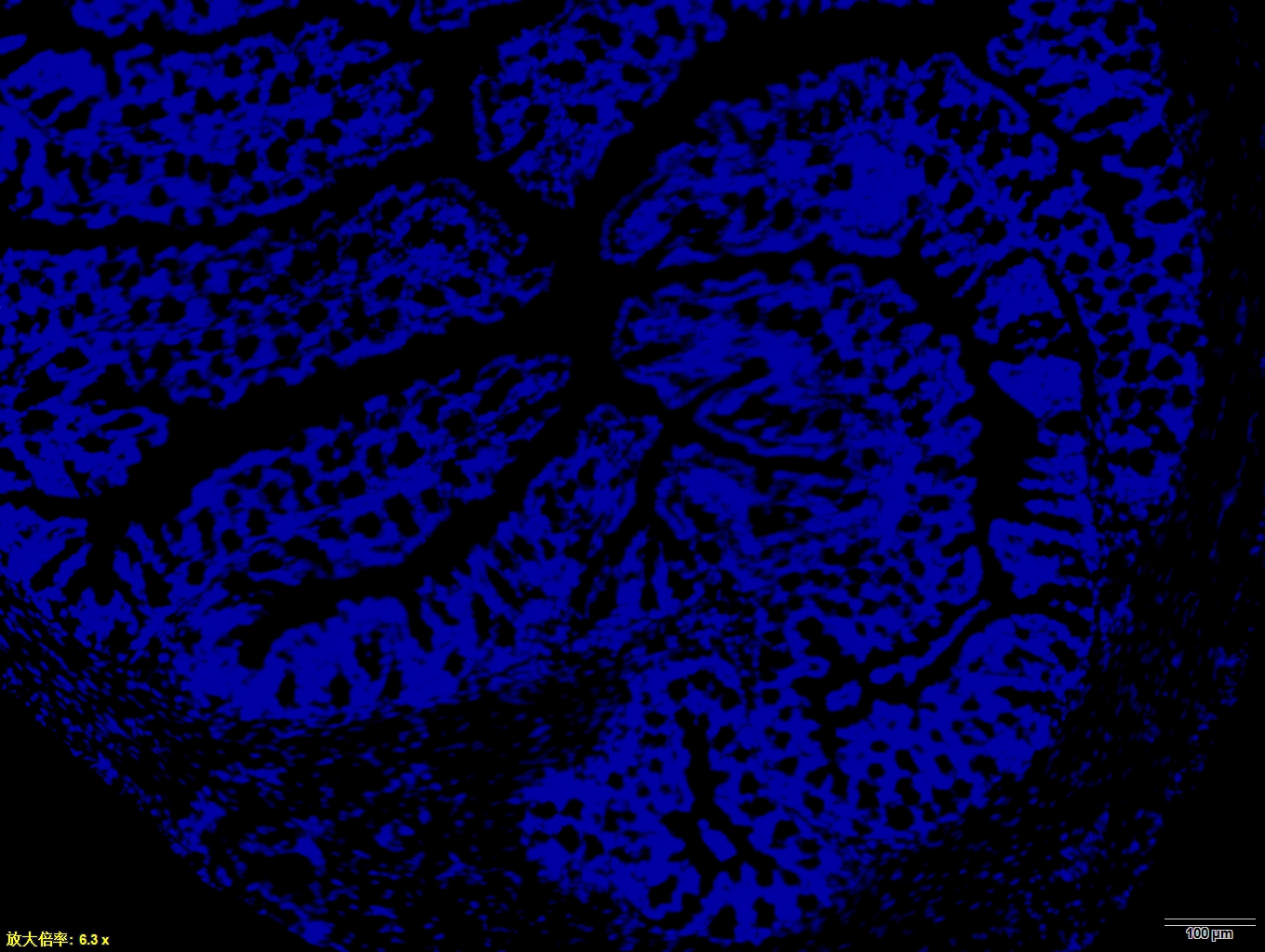

Supplement: Supplementary file 2 [file Data_Sheet_2.zip › Figure 6/DSS+MOLP-L Occludin DAPI .jpg]

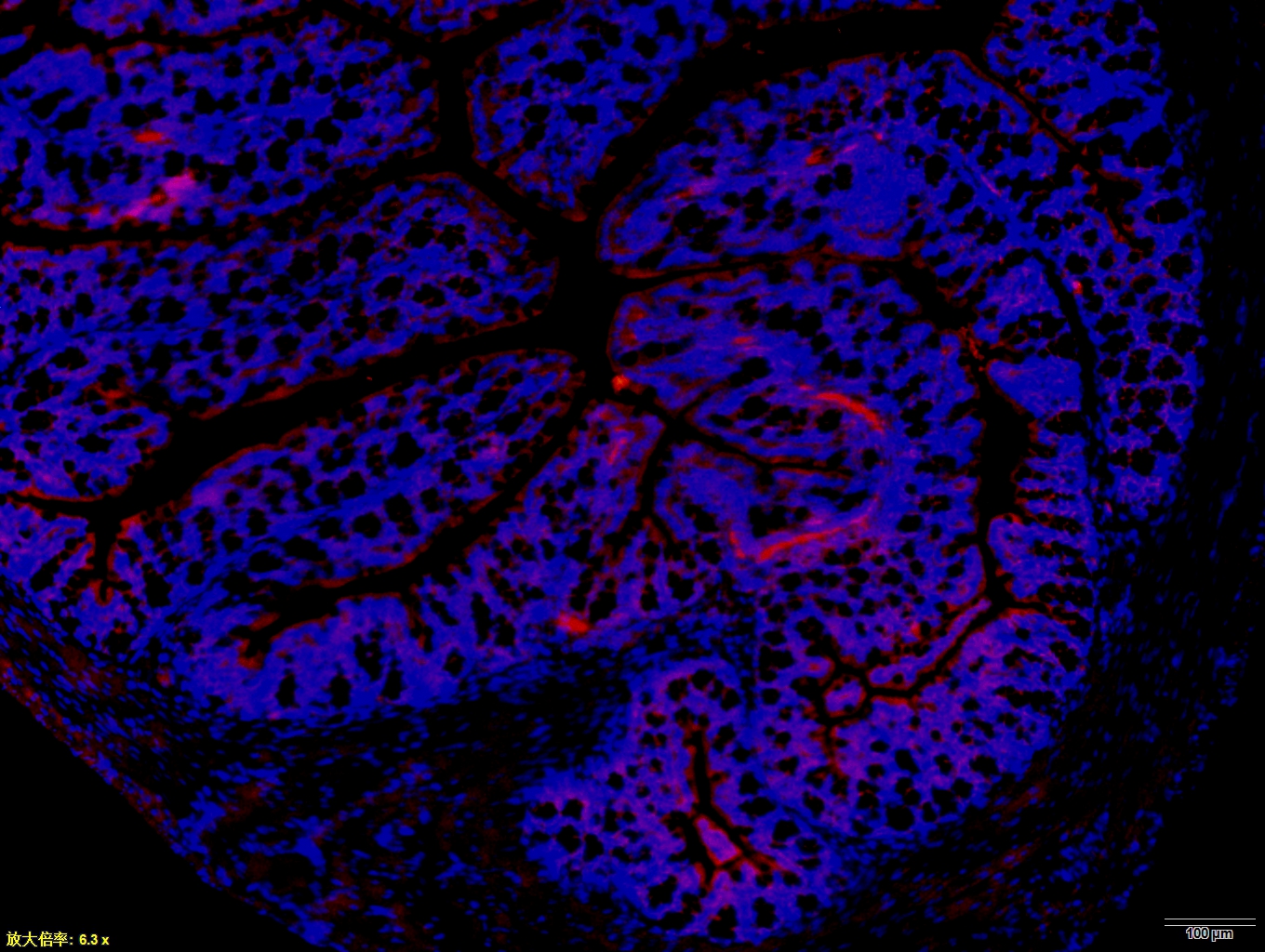

Supplement: Supplementary file 2 [file Data_Sheet_2.zip › Figure 6/DSS+MOLP-L Occludin MERGE .jpg]

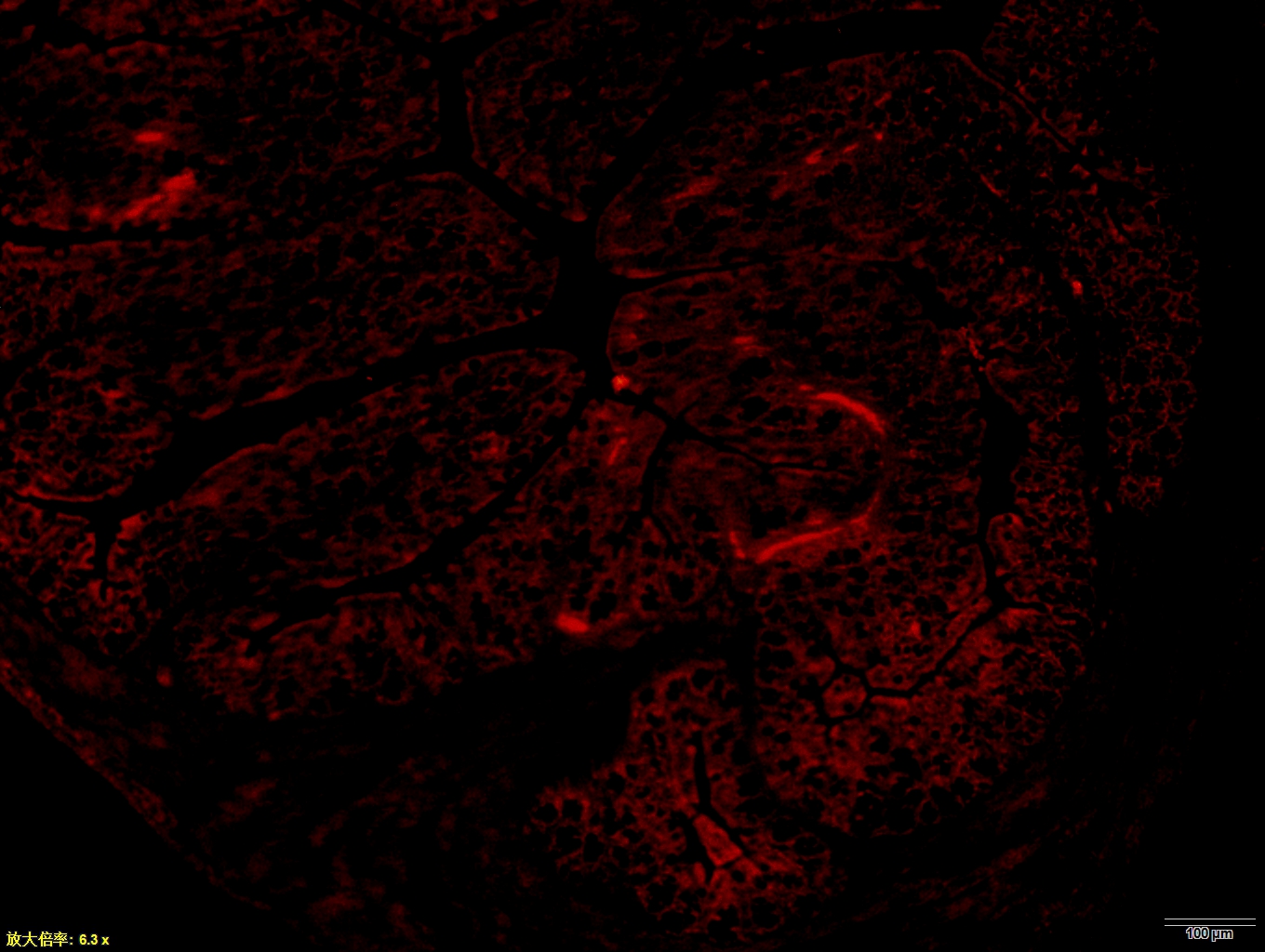

Supplement: Supplementary file 2 [file Data_Sheet_2.zip › Figure 6/DSS+MOLP-L Occludin.jpg]

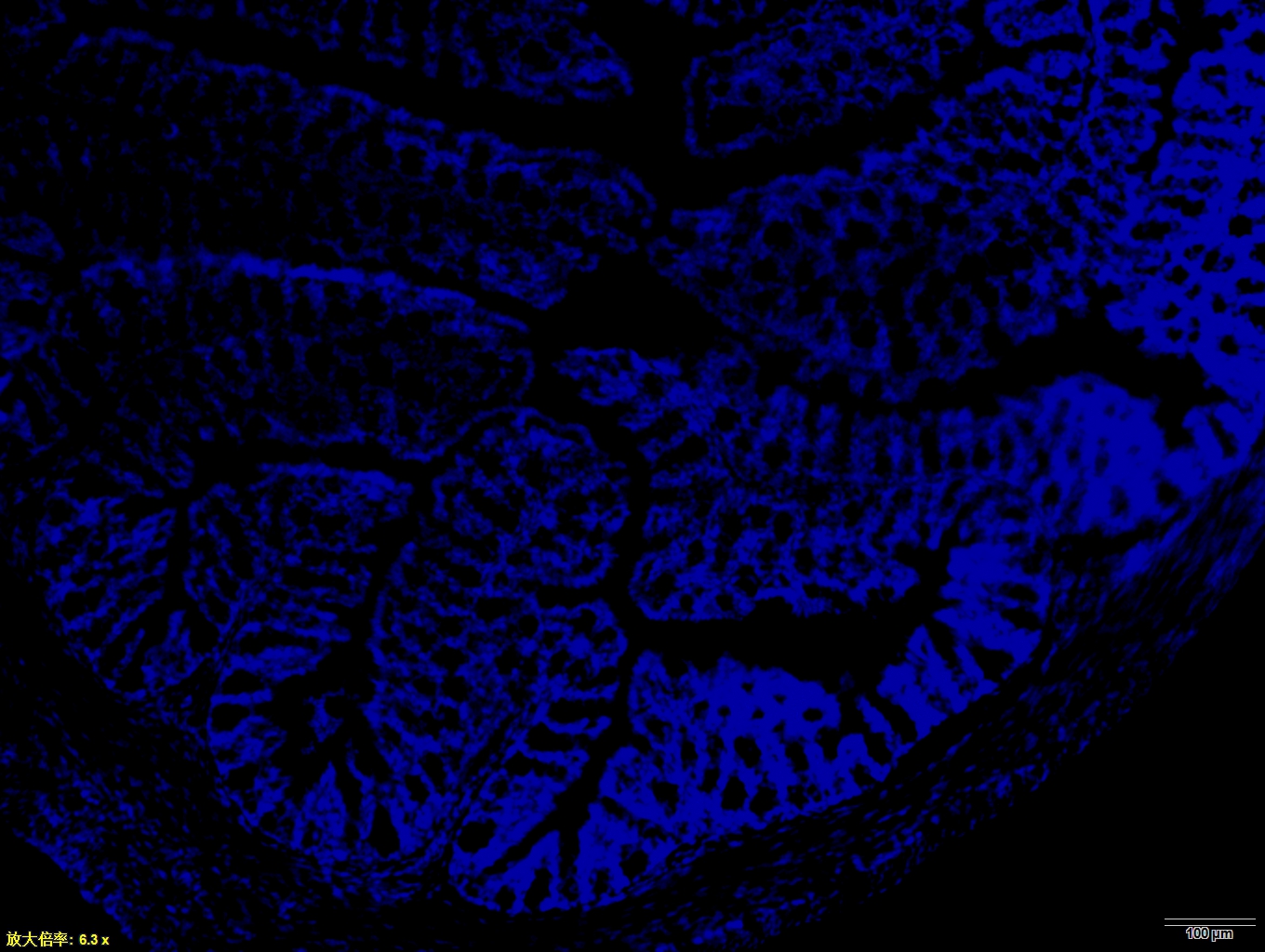

Supplement: Supplementary file 2 [file Data_Sheet_2.zip › Figure 6/DSS+MOLP-L ZO-1 DAPI.jpg]

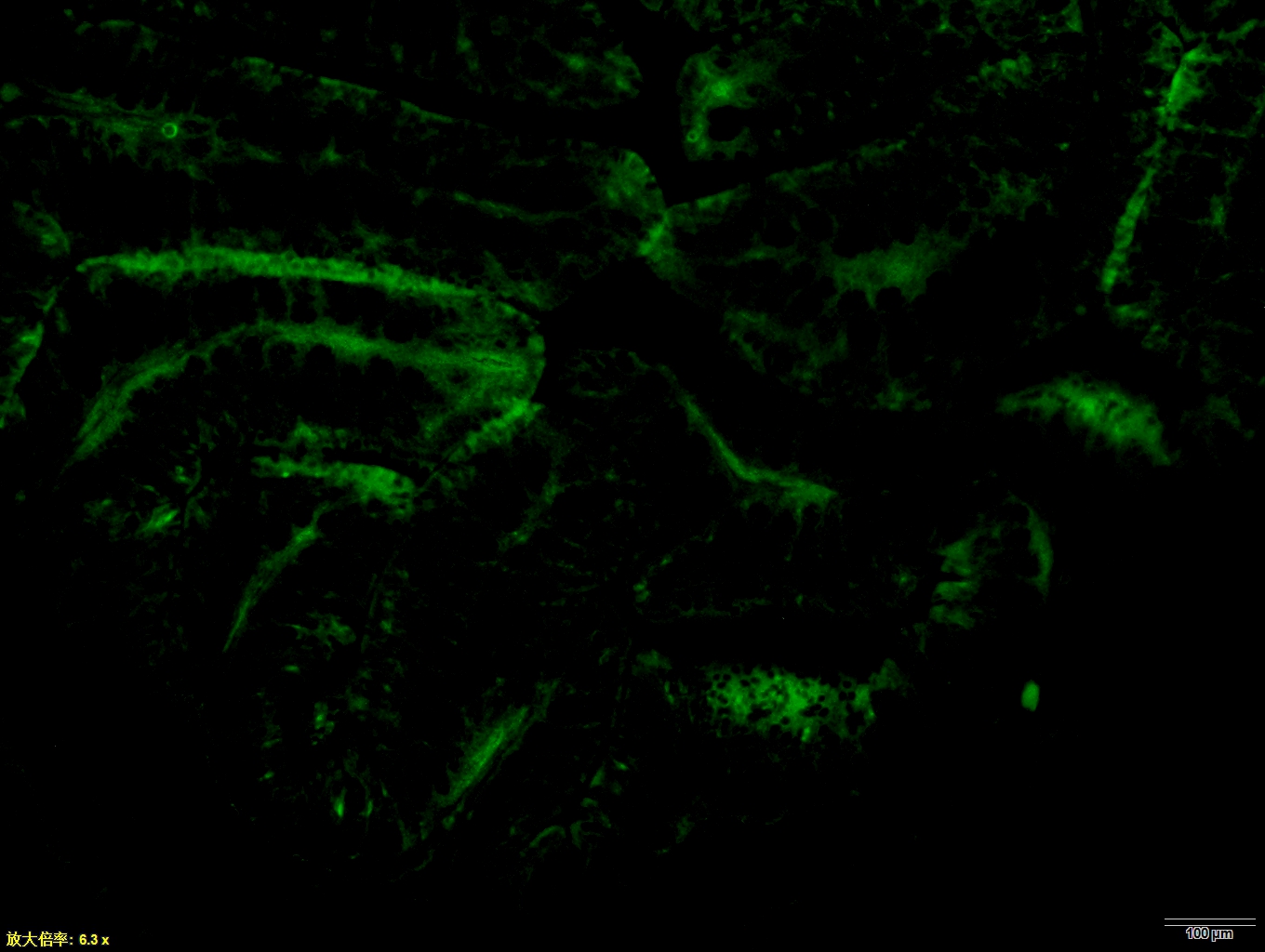

Supplement: Supplementary file 2 [file Data_Sheet_2.zip › Figure 6/DSS+MOLP-L ZO-1.jpg]

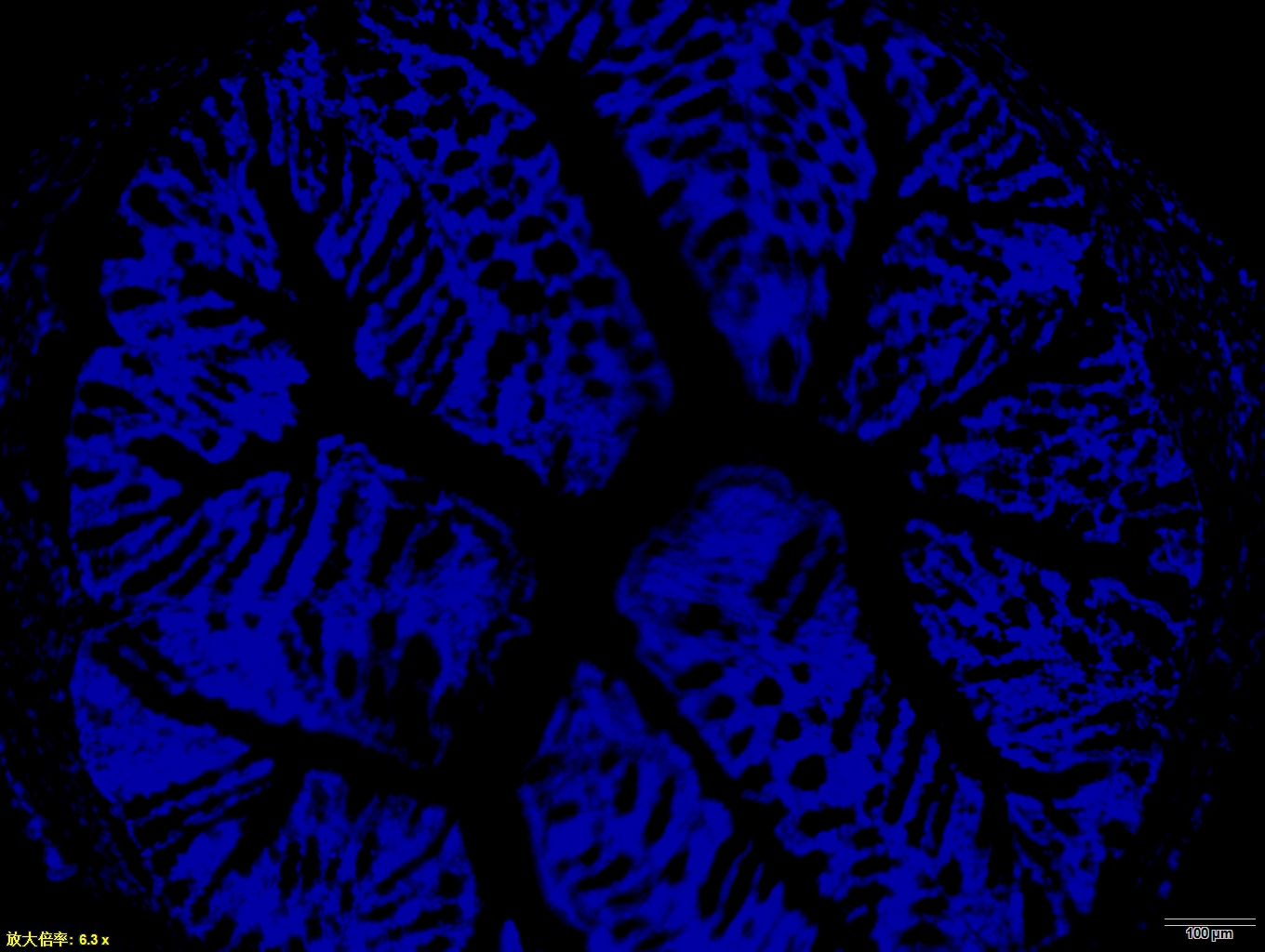

Supplement: Supplementary file 2 [file Data_Sheet_2.zip › Figure 6/DSS+MOLP-M Occludin DAPI.jpg]

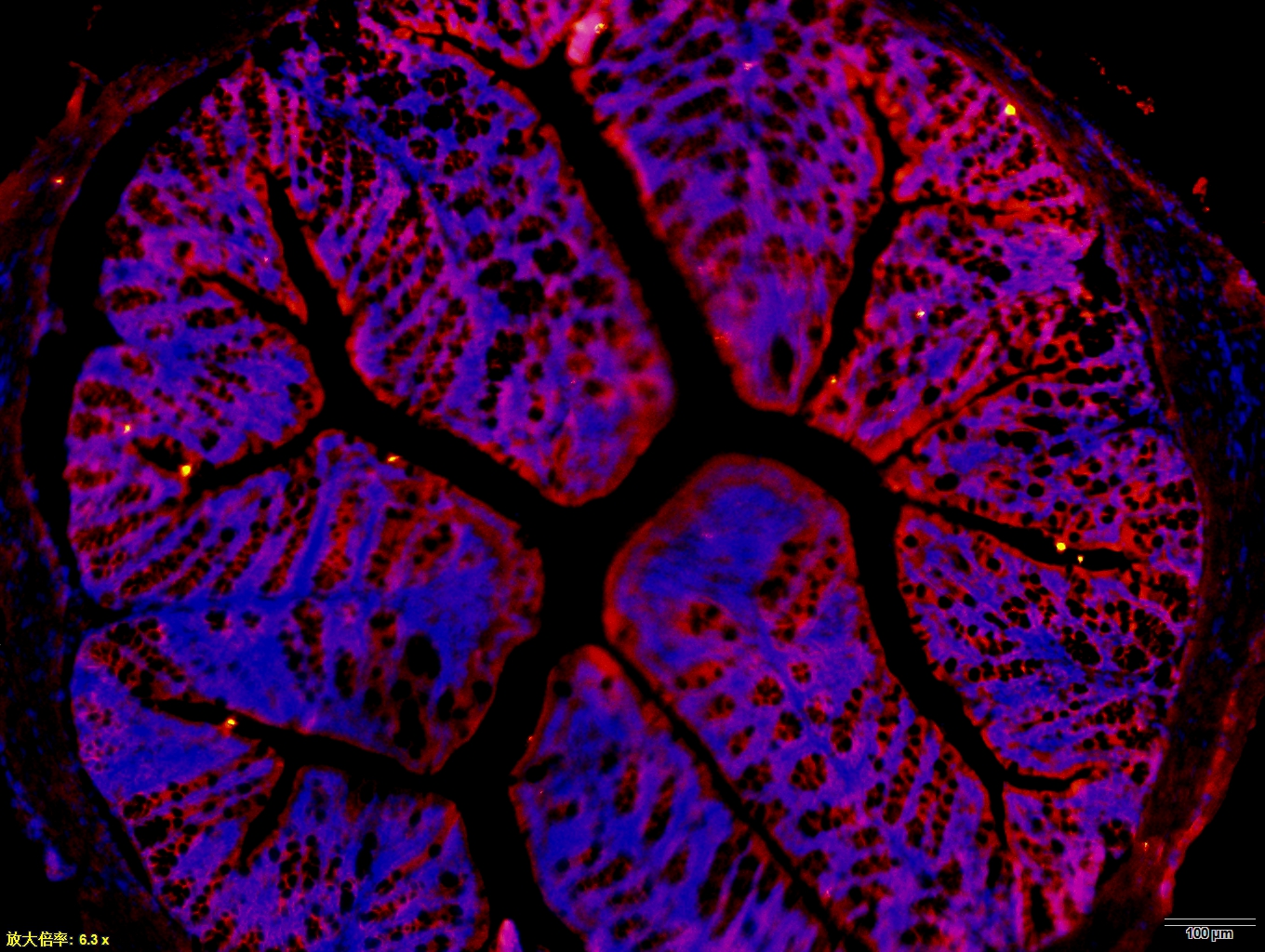

Supplement: Supplementary file 2 [file Data_Sheet_2.zip › Figure 6/DSS+MOLP-M Occludin merge.jpg]

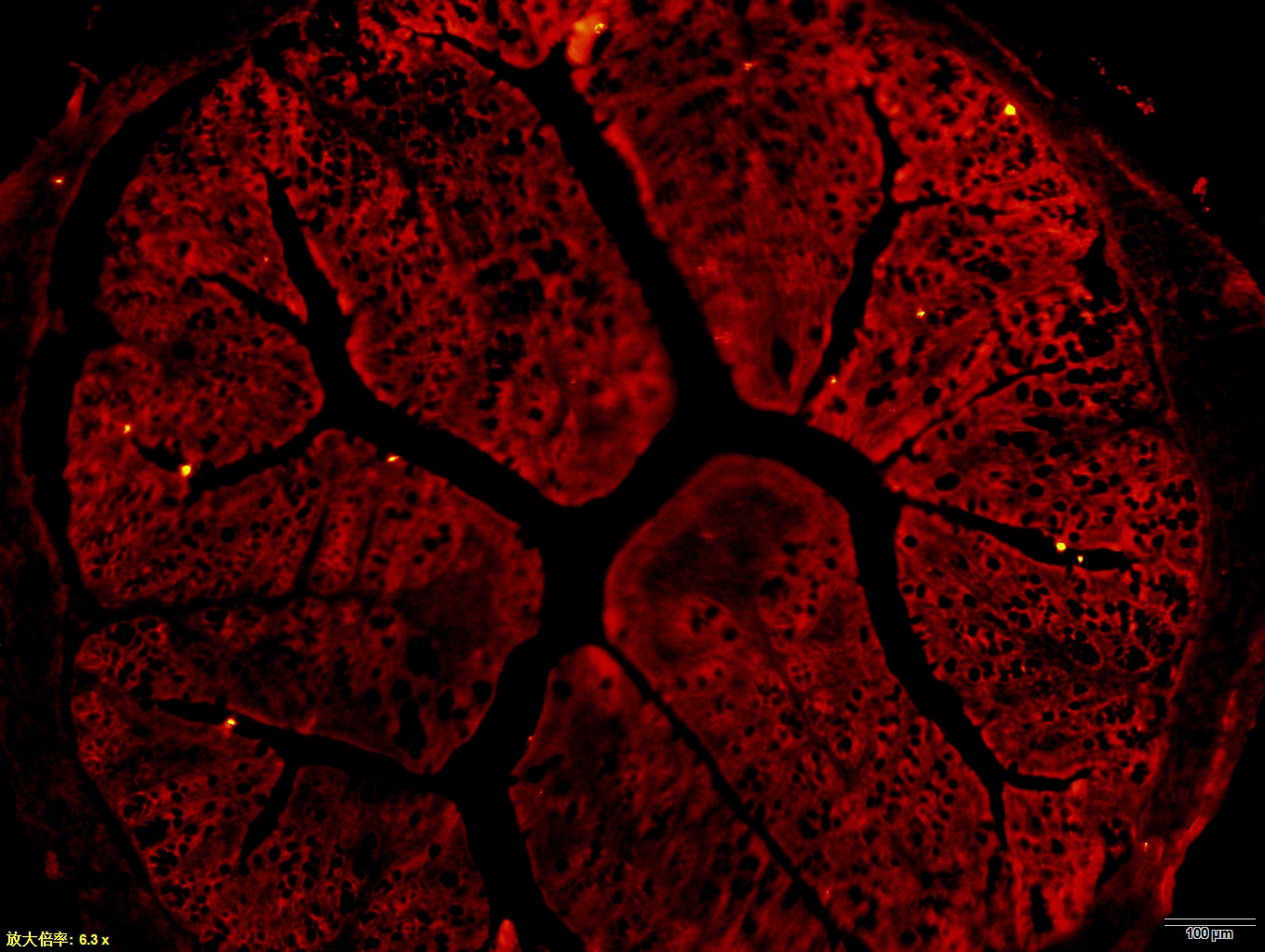

Supplement: Supplementary file 2 [file Data_Sheet_2.zip › Figure 6/DSS+MOLP-M Occludin.jpg]

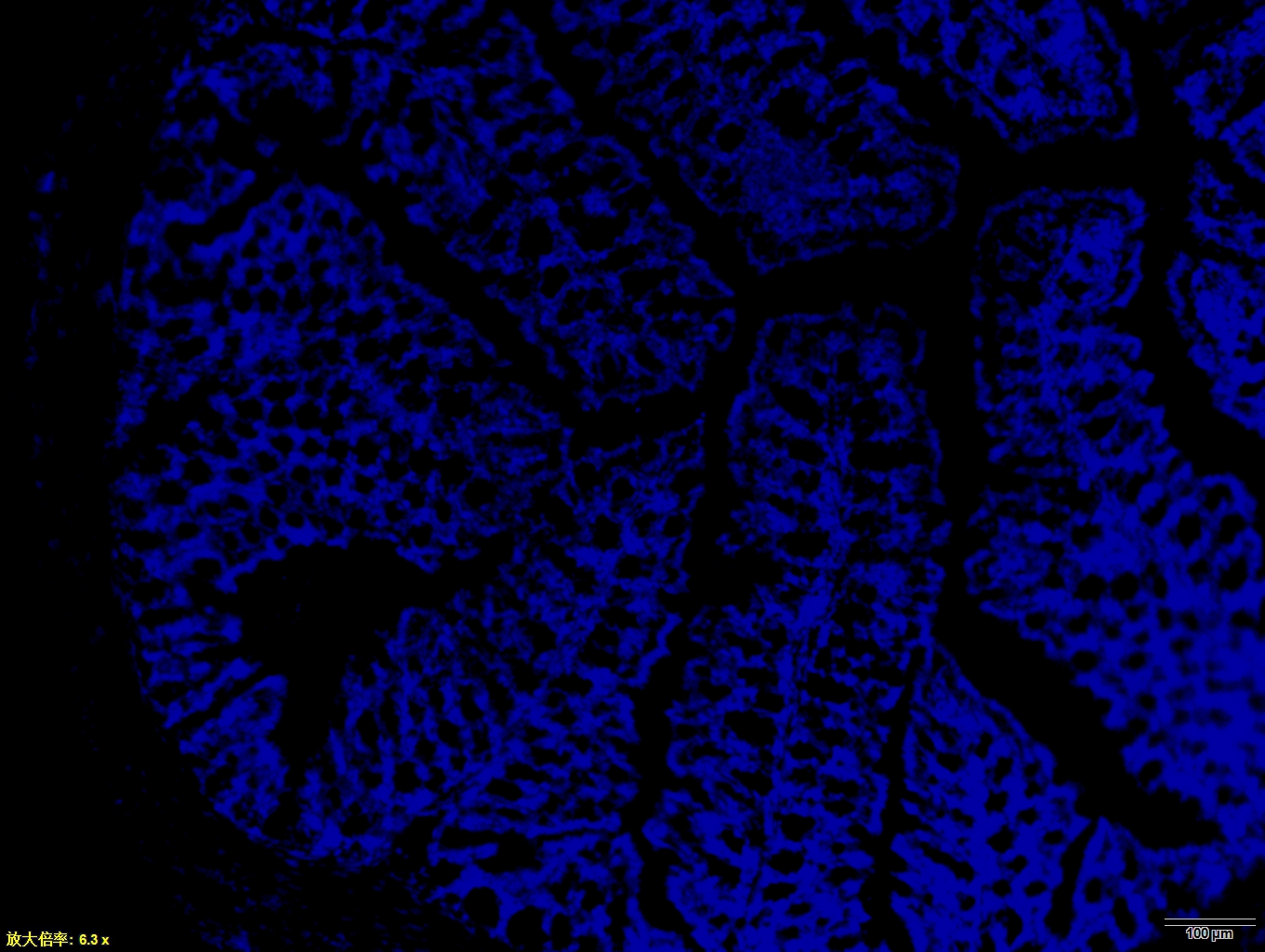

Supplement: Supplementary file 2 [file Data_Sheet_2.zip › Figure 6/DSS+MOLP-M ZO-1 DAPI.jpg]

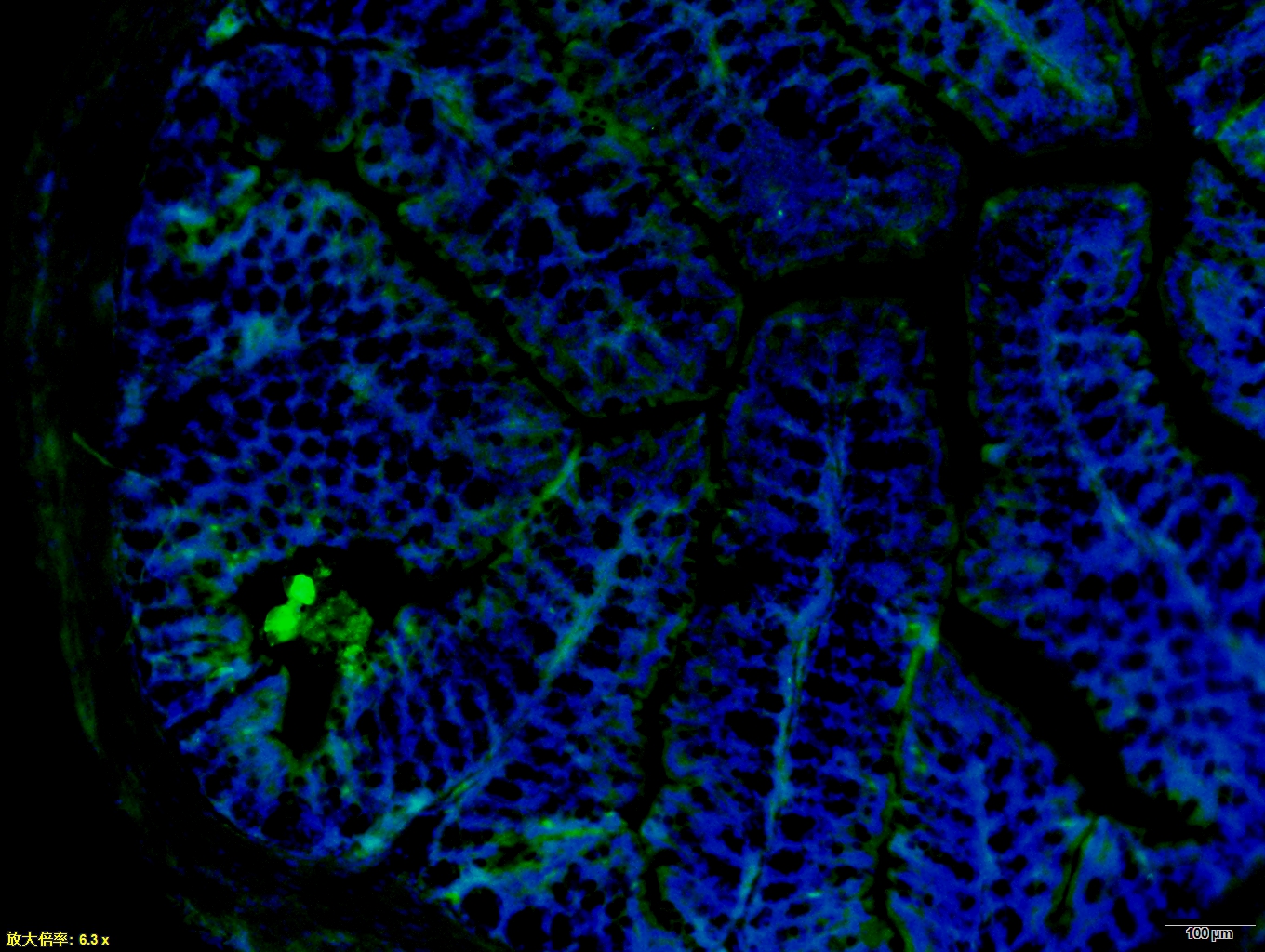

Supplement: Supplementary file 2 [file Data_Sheet_2.zip › Figure 6/DSS+MOLP-M ZO-1 MERGE.jpg]

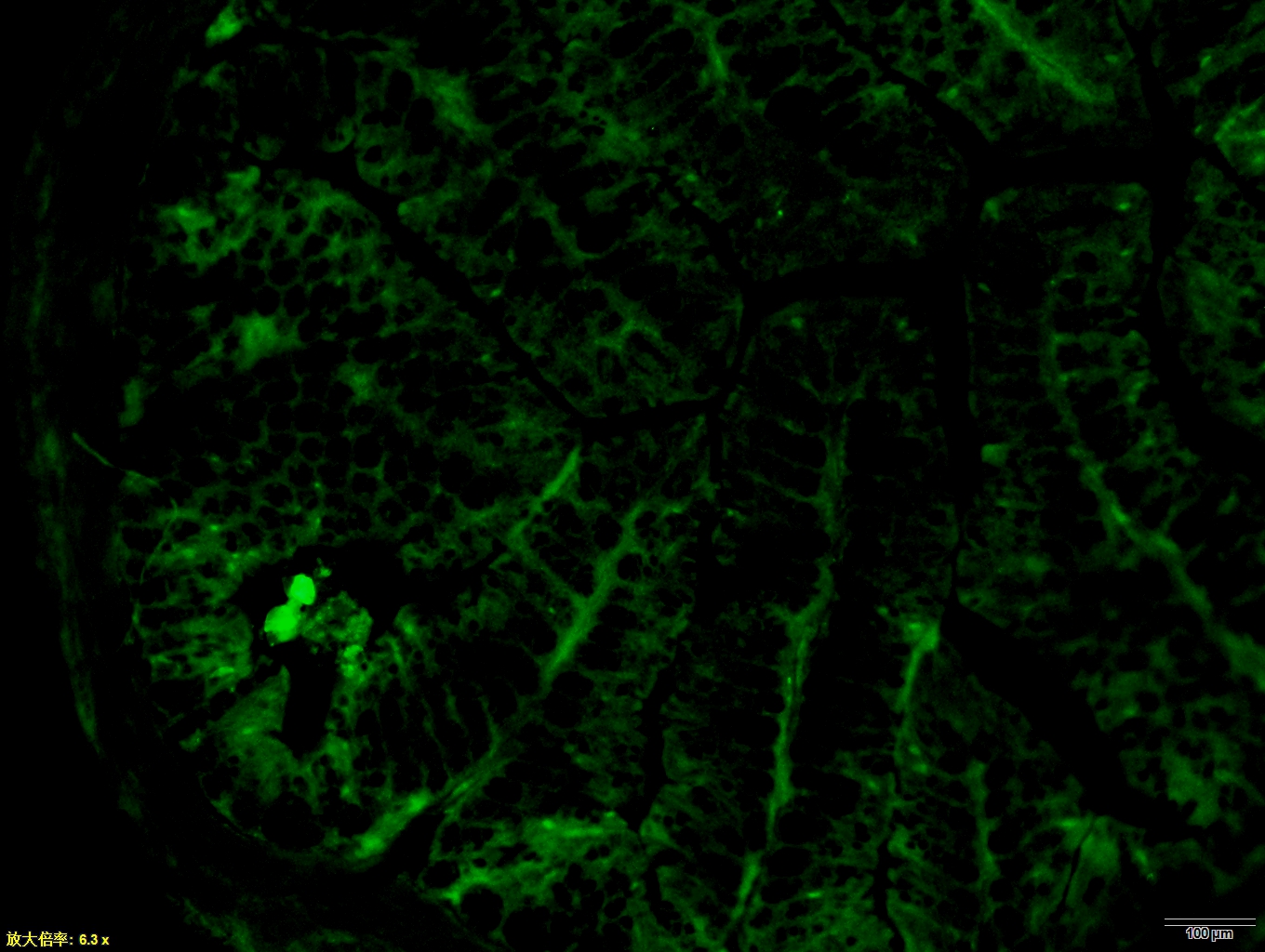

Supplement: Supplementary file 2 [file Data_Sheet_2.zip › Figure 6/DSS+MOLP-M zo-1.jpg]

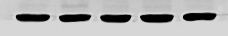

Supplement: Supplementary file 2 [file Data_Sheet_2.zip › Figure 5/ACTIN .jpg]

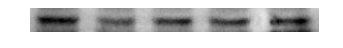

Supplement: Supplementary file 2 [file Data_Sheet_2.zip › Figure 5/IKB.jpg]

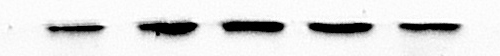

Supplement: Supplementary file 2 [file Data_Sheet_2.zip › Figure 5/MYD88 1.jpg]

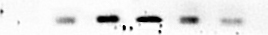

Supplement: Supplementary file 2 [file Data_Sheet_2.zip › Figure 5/p-ikba .jpg]

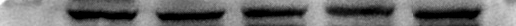

Supplement: Supplementary file 2 [file Data_Sheet_2.zip › Figure 5/p65 .jpg]

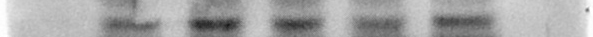

Supplement: Supplementary file 2 [file Data_Sheet_2.zip › Figure 5/pp65 .jpg]

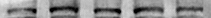

Supplement: Supplementary file 2 [file Data_Sheet_2.zip › Figure 5/TLR4 .jpg]
